# Development of Connectivity in a Motoneuronal Network in *Drosophila* Larvae

**DOI:** 10.1016/j.cub.2014.12.056

**Published:** 2015-03-02

**Authors:** Louise Couton, Alex S. Mauss, Temur Yunusov, Soeren Diegelmann, Jan Felix Evers, Matthias Landgraf

**Affiliations:** 1 Department of Zoology, University of Cambridge, Cambridge CB2 3EJ, UK; 2 Centre for Organismal Studies, Ruprecht-Karls-Universität, 69120 Heidelberg, Germany

## Abstract

**Background:**

Much of our understanding of how neural networks develop is based on studies of sensory systems, revealing often highly stereotyped patterns of connections, particularly as these diverge from the presynaptic terminals of sensory neurons. We know considerably less about the wiring strategies of motor networks, where connections converge onto the dendrites of motoneurons. Here, we investigated patterns of synaptic connections between identified motoneurons with sensory neurons and interneurons in the motor network of the *Drosophila* larva and how these change as it develops.

**Results:**

We find that as animals grow, motoneurons increase the number of synapses with existing presynaptic partners. Different motoneurons form characteristic cell-type-specific patterns of connections. At the same time, there is considerable variability in the number of synapses formed on motoneuron dendrites, which contrasts with the stereotypy reported for presynaptic terminals of sensory neurons. Where two motoneurons of the same cell type contact a common interneuron partner, each postsynaptic cell can arrive at a different connectivity outcome. Experimentally changing the positioning of motoneuron dendrites shows that the geography of dendritic arbors in relation to presynaptic partner terminals is an important determinant in shaping patterns of connectivity.

**Conclusions:**

In the *Drosophila* larval motor network, the sets of connections that form between identified neurons manifest an unexpected level of variability. Synapse number and the likelihood of forming connections appear to be regulated on a cell-by-cell basis, determined primarily by the postsynaptic dendrites of motoneuron terminals.

## Highlights

• Growing motoneurons consolidate synapses with existing presynaptic partners
• Motoneuron dendritic arbors are active parties in setting connectivity patterns
• Cell-type-specific features coexist with variations at the individual cell level
• Motoneuron wiring strategies may contrast with those of sensory neurons

## Teaser

The development of neural connections has mainly been studied in the context of sensory networks. Couton et al. explore how, in a motor network, synaptic connections change during development, finding that connectivity patterns show considerable variability between cells and that motoneuron dendrites play an active role in shaping these.

## Introduction

Much of our current view of how sets of synaptic connections form and change during nervous system development is derived from studies of sensory systems [1–6]. The connections that sensory neurons form are often tightly constrained, enabling the formation of accurate sensory maps, with numbers and distributions of synapses appropriate for network operation [7, 8]. Connectivity at lower-order synapses of the network can be almost invariant and cell autonomously specified. For example, *Drosophila* photoreceptor neurons reproducibly form ∼50 synapses with specific postsynaptic lamina cells, irrespective of photoreceptor function or visual system defects [9, 10]. At higher-order synapses, in contrast, connectivity can be rather variable, reflecting both experience-dependent plasticity and distinct wiring strategies [11, 12]. For example, randomized connections in the mushroom body are thought to maximize coding space [13, 14].

Here, we focus on the much less well-explored development of connectivity within a motor network. Motor systems manifest a great deal of flexibility, including their ability to adjust to changes in muscle size with growth and exercise, thus maintaining the capacity to trigger effective muscle contractions. This has been most extensively studied at the neuromuscular junction where the growth of the presynaptic terminal is matched with that of the postsynaptic muscle, regulated by muscle-derived retrograde signals [15, 16]. In addition, motoneurons also adjust centrally through changes in the size and connectivity of their dendritic arbors [17].

To investigate patterns of connectivity in a motor network and how these change as the animal develops and grows, we used the *Drosophila* larva as a model. We developed a paradigm for studying identified partner neurons at the level of individual synaptic sites across different developmental stages. We asked the following questions: (1) How does connectivity change as the motor network develops? (2) How reproducible or variable are the sets of connections that form? (3) Is there evidence of synaptic patterning information residing with the presynaptic or postsynaptic partner? We show that from hatching to later larval stages, existing connections are progressively consolidated by addition of synapses. We find that while patterns of connections are specific to each motoneuron type, considerable variability remains. Moreover, connectivity appears to be set on a cell-by-cell basis by the dendritic arbors of motoneurons, and dendritic positioning is a determinant of the connections that motoneurons make. Together, these findings argue in favor of a flexible regulation of connectivity in the assembly of the larval crawling circuit.

## Results

### Imaging Putative Synaptic Connections between Identified Neurons in a Developing Motor System

**Figure 1:**
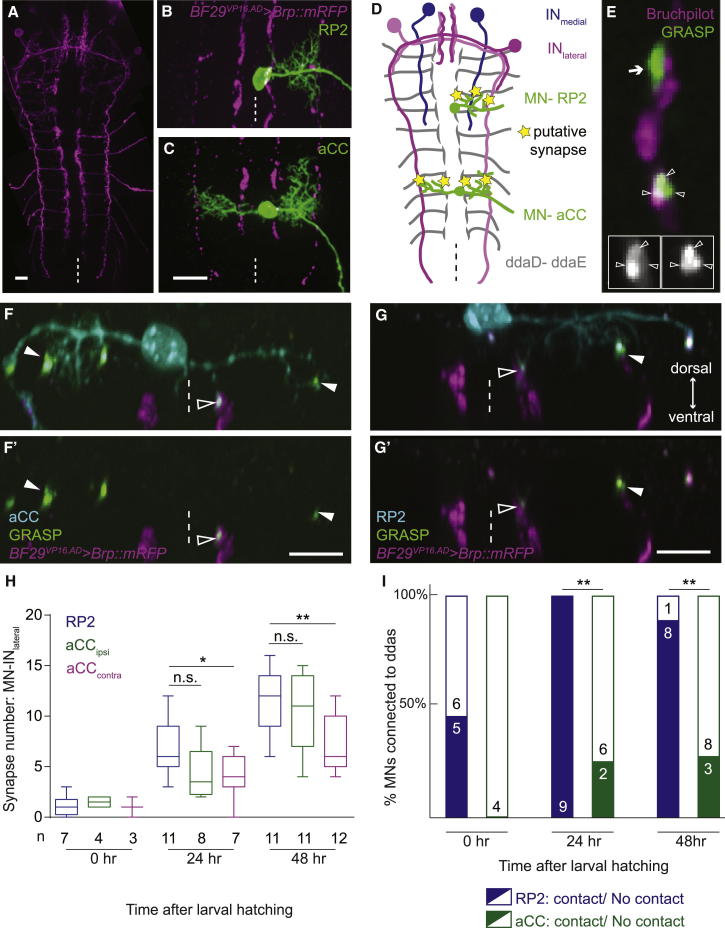
Genetic Tools to Differentially Label Partner Neurons Reveal a Motoneuron-Specific Pattern of Connectivity during Development (A and D) Intersection of the split-Gal4 line *BF29*^*VP16.AD*^ with *Cha(7.4kb)*^*Gal4.DBD*^^J8A1^ targets Gal4 activity to two pairs of descending interneurons, namely a lateral (IN_lateral_) and a medial (IN_medial_) interneuron on each side of the midline, as well as to the segmentally repeated dda sensory neurons (ddaD and ddaE). (B–D) Two motoneurons (aCC and RP2) that make connections with these interneurons are visualized with a LexA-Flpout system. (E) Synaptic contacts between partner neurons (arrowheads) are identified by co-localization of the presynaptic active zone marker Bruchpilot (Brp) and the cell-cell contact reporter (GRASP). The arrow points to a contact region between partner neurons that is devoid of presynaptic Brp::mRFP and therefore not considered a putative synaptic site. Insets show the synapse area as separate channels. (F–G′) Cross section views of nerve cords showing the location of aCC and RP2 motoneurons, relative to their presynaptic sites from their common partners, IN_lateral_ (filled arrowheads) and dda terminals (open arrowheads). (F′) and (G′) show the GRASP and Brp channels separately. (H and I) Quantification of the development of connections between the aCC and RP2 motoneurons and the IN_lateral_ (H) and ddas (I) during larval life. Boxplots show the median of the distribution (middle line), the 75^th^ (upper limit of box) and 25^th^ (lower limit of box) percentile; whiskers indicate the highest and lowest value of each dataset. Each of the dendritic arbors shows a significant increase in connectivity with the IN_lateral_ over time: ANOVA (p values < 0.01) with post tests for linear trend: p < 0.0001 for RP2 and aCC ipsilateral; p = 0.0025 for aCC contralateral arbor. RP2 arbors had a significantly different number of synapses with the IN_lateral_ at 24 hr and 48 hr as compared to aCC contralateral arbors: ANOVA (p values = 0.06 and 0.01) with uncorrected Fisher’s least significant difference test, ^∗^p = 0.0415 and ^∗∗^p = 0.0083, respectively. (I) Motoneuron-dda connectivity or absence thereof is shown. RP2 and aCC differ significantly in their sensory-motor connectivity, at 24 hr and 48 hr (Fisher’s exact test, ^∗∗^p = 0.0023 and ^∗∗^p = 0.0098, respectively). Anterior is up in (A)–(E); dorsal is up in (F)–(G′). Dashed line represents the midline. Scale bars represent 10 μm, except in (E), where each inset is 5 × 5 μm. See Figures S1–S3 for tests of GRASP and Brp::mRFP for reporting on synapses.

To study the emergence of synaptic connectivity in a motor network as it develops, we generated genetic tools for reliably visualizing and manipulating identified, connecting neurons in the *Drosophila* larval nerve cord. For pre-motor partner neurons, we fractionated through an intersectional “split-Gal4” enhancer trap screen [18] the set of cholinergic interneurons and sensory neurons, which provide the synaptic drive to motoneurons in this system [19]. From >3,000 lines, we identified those with sparse expression and terminations in the motor neuropile. Single motoneurons (“aCC” and “RP2”) were visualized via a LexA/LexAOp and FLP recombinase-based quaternary system [20] (see Supplemental Experimental Procedures). To resolve synaptic sites, we combined the presynaptic active zone marker *UAS-brp::mRFP* [21] with the GFP reconstitution across synaptic partners (GRASP)-based reporter for cell-cell contacts [22]. Brp::mRFP-positive presynaptic specializations that coincide with physical appositions of presynaptic and postsynaptic membranes, as reported by GRASP, were scored as putative synapses (Figures 1A–1G′; see Figures S1–S3 and Movie S1 for technical validation). We thus charted patterns of connectivity during larval development, from 0 hr after larval hatching (ALH) to the third instar stage (48 hr ALH), between the aCC and RP2 motoneurons and some of their presynaptic partners, made accessible to analysis by the Split-Gal4 line *BF29*^*VP16.AD*^: two intersegmental descending interneurons and the ddaD and ddaE proprioceptive sensory neurons [23] (Figures 1H and 1I) (see Supplemental Experimental Procedures for details).

We focused on the lateral interneuron (IN_lateral_) within the *BF29*^*VP16.AD*^ expression pattern; its axon descends contralaterally from the sub-esophageal ganglion to segment A8 and forms putative en passant synapses with intersegmental nerve motoneurons. In mid-abdominal segments (A2–A6), the number of putative synaptic connections between this IN_lateral_ and the RP2 motoneuron increases steadily with developmental time from an average of 0.86 ± 0.26 at 0 hr ALH to 6.73 ± 0.78 at 24 hr ALH to 11.09 ± 0.97 at 48 hr ALH (n = 7–11) (Figure 1H). This developmental increase in synapse number is compatible with electrophysiological recordings from these motoneurons [17]. IN_lateral_ axons also form putative synapses with the two dendritic sub-arbors of the aCC motoneuron. The larger ipsilateral arbor, located on the same side as the aCC soma, receives more putative synapses from the IN_lateral_ than the smaller sub-arbor on the contralateral side (Figure 1H). Both RP2 and aCC project to dorsal body wall muscles. To extend these observations to motoneurons that innervate ventral muscles, we manually labeled RP3 motoneurons with the lipophilic tracer dye DiD and charted co-localization with IN_lateral_ Brp::mRFP sites as putative connections. Here, too, we found that the number of putative connections between this pair of neurons increases with developmental time, from 1 synapse (±0, n = 3) at 0 hr ALH to an average of 3.6 synapses (±0.4, n = 5) at 24 hr ALH.

**Figure 2:**
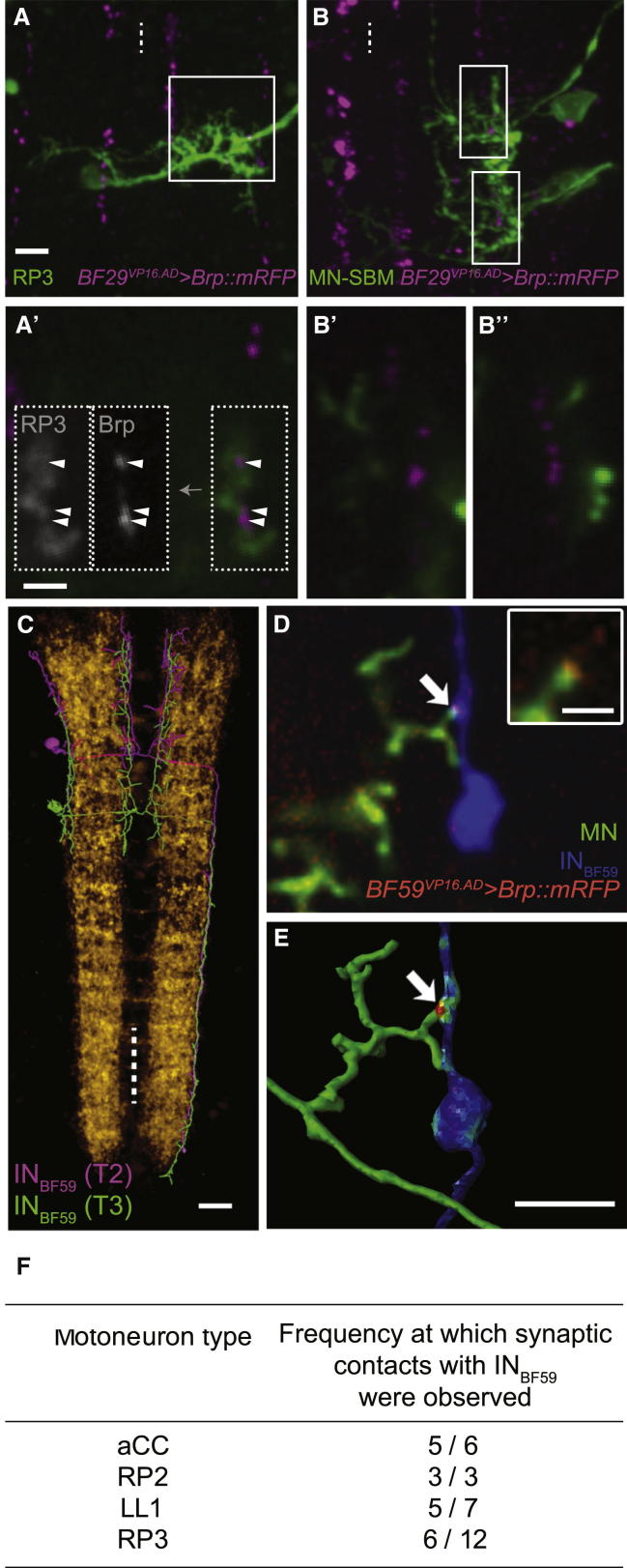
Variable Connectivity between Intersegmental Interneurons and Motoneurons (A–B′′) Comparison of connectivity between an interneuron from the split-Gal4 expression line *BF29*^*VP16.AD*^ (IN_lateral_) and two motoneuron types: RP3, innervating longitudinal muscles, and the motor neuron of the segment border muscle (MN-SBM), innervating the transverse segment border muscle (SBM). Z projections of confocal image sub-stacks show presynaptic sites reported by *UAS-brp::mRFP* (magenta) and motoneurons manually labeled with DiD (green). (A′), (B′), and (B′′) show the insets in (A) and (B) in more detail, as single confocal planes. The gray level insets in (A′) show the Brp::mRFP and DiD motorneuron channels separately. Although RP3 and the MN-SBM cover similar dendritic areas around the IN_lateral_, RP3 shows putative contacts (arrowheads), whereas the MN-SBM does not. (C) First instar larval nerve cord with neuropil visualized with alpha-Bungarotoxin-Alexa Fluor 488 (yellow) and two reconstructions of intersegmental interneurons from the split-Gal4 expression line *BF59*^*VP16.AD*^ intersected with *Cha(7.4kb)*^*Gal4.DBD J8A1*^, shown in thoracic segments T2 (magenta) and T3 (green). (D and E) Putative synaptic contacts between motoneurons and these intersegmental interneurons (IN_BF59_) at larval hatching. Interneurons expressing *UAS-brp::mRFP* (red) were manually labeled with DiO (blue), and motoneurons were visualized with DiD (green). (D) Single confocal optical section. (E) Reconstructed interneuron (blue) and partially reconstructed dendritic arbor of motoneuron (MN, green) where a site of likely physical overlap coinciding with a presynaptic site is highlighted red (arrow). The inset in (D) is a single confocal section showing the overlap in more detail in the motoneuron (green) and presynaptic marker (red) channels. (F) Table of frequencies at which synaptic contacts with BF59^VP16.AD^ interneurons were observed for different motoneuron types. Anterior is up. Ventral midline is indicated by dashed line. Scale bars of (A), (B), and (C) represent10 μm; scale bars of (A′), (B′), (B′′), (D), and (E) represent 5 μm; the scale bar of (D) inset represents 1 μm.

Next, we looked at cell-type-specific differences in connectivity. These are most evident in the likelihood with which the RP2 and aCC motoneurons receive putative synapses from the ddaD and ddaE sensory terminals (the high density of Brp::mRFP puncta in these sensory terminals prevents resolution of individual puncta; Figures 1F, 1G, and 1I). As larvae develop, this sensory-motor connection becomes increasingly frequent, although throughout aCC, motoneurons have a significantly lower probability than RP2 of forming putative synapses with these dda sensory terminals (Figure 1I). In addition, we found that motoneurons such as RP3, which are similar in operation to RP2 and aCC, i.e., in innervating longitudinal body wall muscles, also form putative synapses with the presynaptic IN_lateral_, while motoneurons innervating antagonistic transverse muscles [24] do not, even though their dendrites arborize within reach of the IN_lateral_ axon (Figures 2A–2B′′). For another pre-motor interneuron, IN_BF59_, labeled with the *BF59*^*VP16.AD*^ expression line (Figure 2C), we resolved single cells by injecting IN_BF59_ interneurons expressing *UAS-brp::mRFP* with the lipophilic tracer dye, Neuro-DiO, and different motoneurons with the spectrally distinct DiD. Co-localization of these three markers (Neuro-DiO, Brp**::**mRFP, and DiD) was taken as indicative of a putative synapse (Figures 2C–2F). The data suggest that different motoneurons, projecting to dorsal (aCC, RP2), lateral (MN-LL1), and ventral (RP3) muscles, may have different likelihoods of contacting the IN_BF59_.

In summary, in this motor network, the number of putative synapses between partner neurons generally increases as the network matures and the animal grows. Different motoneurons have different likelihoods of forming synapses with the same sets of presynaptic sensory neurons. Such qualitative differences are suggestive of motoneuron-type-specific regulation of connectivity.

### Connectivity between Identified Neurons Shows Considerable Levels of Variability

**Figure 3:**
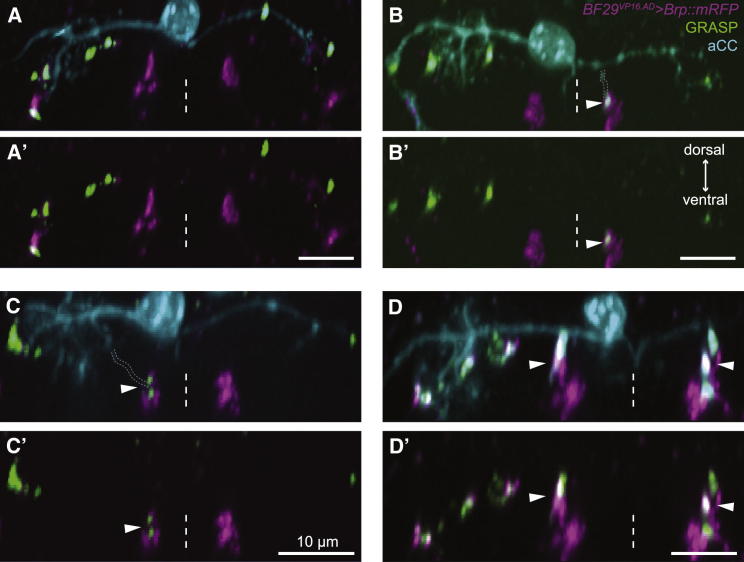
aCC Motor-Sensory Neuron Connections Are Achieved through Different Routes (A–D′) Cross-section views of nerve cords showing the different configurations of aCC-dda connections (arrowheads): no connection (A), contralateral (B), ipsilateral (C), or bilateral (D). In order to make the distinction among pseudo-colored motoneuron (cyan), GRASP (green), and Brp::mRFP (magenta) easier, the GRASP and Brp channels are also displayed separately in (A′), (B′), (C′), and (D′). In (B) and (C), aCC neurites connecting to the ddas are outlined with dotted lines to show that synaptic dendritic segments do not always originate from the main dendritic tree. Dorsal is up. All scale bars represent 10 μm. Dashed line indicates midline. All data were collected 24 hr ALH.

We were struck by how variable connectivity between identified neurons seems to be. For example, the number of putative synapses between IN_lateral_ and RP2 motoneurons ranged from 0 to 3 at 0 hr ALH and 6 to16 at 48 hr ALH (Figure 1H). Similarly, for the sensory-motor connection, only a fraction of RP2 and aCC motoneurons receive putative synaptic contacts from dda sensory terminals (Figure 1I). Here, differences in connectivity are mirrored by the diverse routes by which individual neurons attain their connections (Figure 3). For instance, aCC motoneurons form putative synaptic connections with dda sensory axon terminals in every possible way: with contralateral, ipsilateral, or both groups of sensory projections, established by different routes, with dendrites from the main arbor or the soma. This shows that postsynaptic dendritic arbors of motoneurons are quite flexible in how they attain connections with presynaptic terminals.

### Local Interactions between Partner Cells Underlie Connectivity Patterns

Next, we inquired into possible causes for the variable connectivity that we see. There is no clear indication that the connectivity we have been able to measure becomes progressively more reproducible as the network matures (Figure 1H). We then asked whether differences in segmental identity contributed to the variability we see. Regression analyses show no statistically significant link between the segmental identity of RP2 and aCC motoneurons and the number of putative synapses that these receive from the IN_lateral_ at 0 hr ALH, 24 hr ALH, or 48 hr ALH (Figure S4).

**Figure 4:**
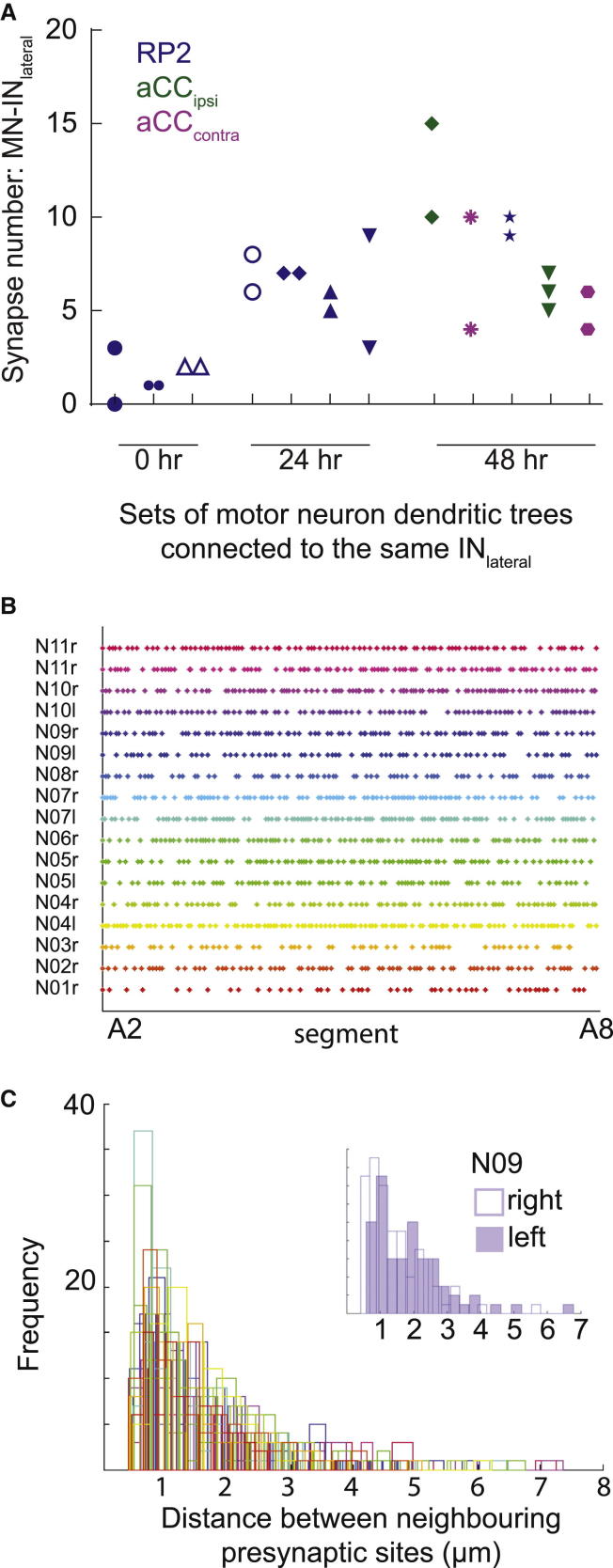
Presynaptic Sites Appear Randomly Distributed along the IN_lateral_ Axon and Do Not Predetermine Connectivity Outcomes (A) Two or more motoneurons of the same kind (RP2, aCC ipsilateral, or aCC contralateral dendritic arbors) can receive different numbers of synaptic connections from the same presynaptic IN_lateral_ axon. The number of synapses does not correlate with the antero-posterior location of the motoneuron (see Figure S4). Observations from different larval stages are displayed. (B) Distribution of *UAS-brp::mRFP*-labeled presynaptic sites along the IN_lateral_ axon traversing segments A2 to A8. Data are shown for 17 neurons from 11 individuals with axons normalized for length. Brp::mRFP puncta distribution along axons was indistinguishable from random (Kolmogorov-Smirnov two-sample test, p = 0.89). (C) Distribution of distances between adjacent presynaptic sites shown in (B) is also indistinguishable from random. Inset in (C): distribution of distances between adjacent presynaptic sites in the IN_lateral_ homologs within one specimen (left is indicated by open bars; right is indicated by filled bars). Differences in local Brp::mRFP puncta densities do not correlate with the number of synapses made onto adjacent motoneurons (see Figure S5).

Next, we considered the effects that local and global network adjustments might have on connectivity. To this end, we focused on pairs of RP2 and aCC neurons located in the same nerve cord and connected to the same IN_lateral_ and asked whether having a common presynaptic partner leads to more similar numbers of synapses formed with the same axon. We find that RP2 and aCC motoneurons can vary substantially in the number of putative connections they receive from the same presynaptic partner (Figure 4A). These data imply that local interactions between individual pairs of neurons, rather than global network effects, might determine the outcome of connectivity.

In summary, these observations suggest that variability in connectivity might be an inherent feature of this motor network, at least for the cells analyzed here.

### Presynaptic Sites Are Randomly Distributed and Not Predictive of Connectivity

Since synapses are the product of interactions between presynaptic and postsynaptic terminals, we wondered whether the variability we observe arises from one or the other synaptic partner. Testing the potential for an instructive role by the presynaptic interneuron, we asked whether there was any pattern to the distribution of presynaptic sites along the IN_lateral_ axon. We found that along the IN_lateral_ axon (segments A2 to A8), the number of presynaptic sites per neuron was highly variable, ranging from 48 to 107 (85 ± 16.8, SD, n = 17). At the same time, the distribution of presynaptic sites and the spacing between these are indistinguishable from random (Figures 4B and 4C). Thus, we see no evidence of positional patterning of en passant presynaptic sites along IN_lateral_ axons, which has been observed in other systems [25–27].

We then asked whether differences in presynapse number could explain the variability in connectivity between different IN_lateral_-motoneuron pairs. To this end, we correlated for each IN_lateral_-motoneuron pair the number of putative synapses formed with the local density of “available” presynaptic Brp::mRFP puncta located within the IN_lateral_ axon along the span of the motoneuron dendritic tree. We found no significant correlation (Pearson’s r = 0.49, p = 0.15, n = 10) (Figure S5). This suggests that, at least in this system, the density of available presynaptic sites is not predictive of how many synaptic connections are formed with the postsynaptic motoneuron. Instead, these data are compatible with a model where the postsynaptic dendritic arbor regulates the number of connections that it forms [28].

### Dendrite Positioning Determines Connectivity

**Figure 5:**
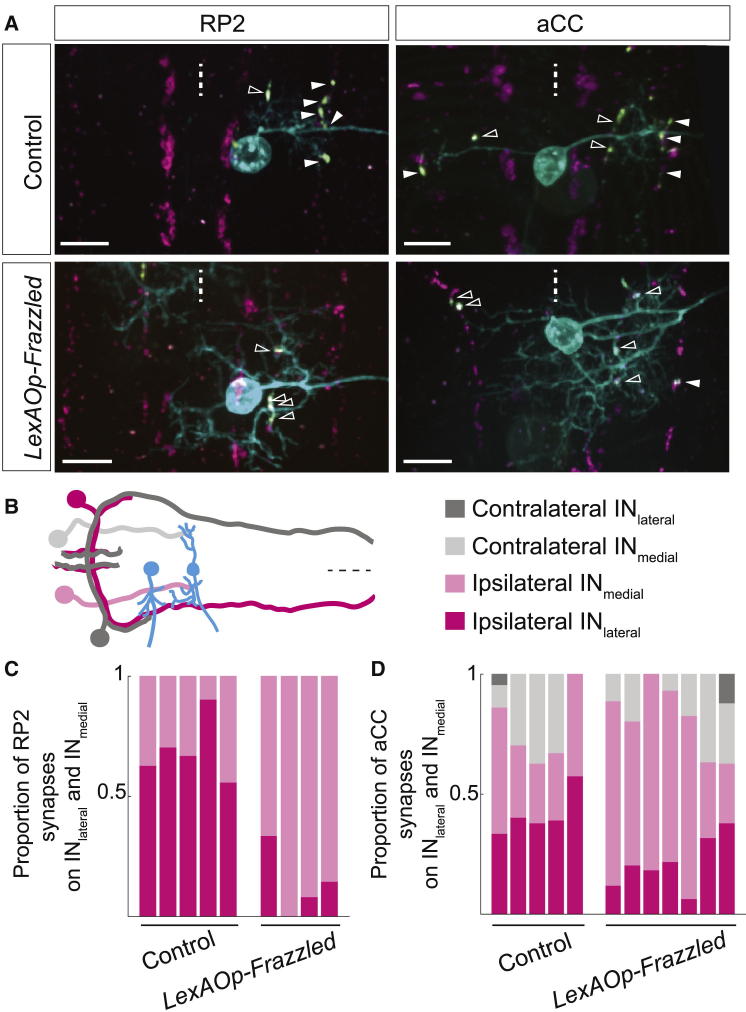
Positioning of Postsynaptic Motoneuron Dendrites Is an Important Factor in Determining the Number of Connections (A) Changing the medio-lateral positioning of motoneuron dendrites by targeted overexpression of the guidance cue receptor Frazzled/DCC shifts the distribution of the dendritic arbor away from the lateral neuropil (where the IN_lateral_ partner axon is located) and toward the midline, closer to the IN_medial_ axon. Magenta indicates *BF29*^*VP16.AD*^ > *brp::mRFP*; cyan indicates motoneurons; green indicates GRASP signal, with arrowheads highlighting contacts with the IN_medial_ (open arrowhead) and IN_lateral_ (filled arrowhead), respectively. (B) Diagram of motoneuron dendrites (cyan) contacting ipsilateral (magenta) and contralateral (gray) IN_lateral_ axons (darker shade) and IN_medial_ axons (lighter shade). (C and D) In thoracic segments, the proportion of synaptic sites that motoneurons make with the IN_lateral_ as compared to the IN_medial_ changes with the repositioning of motoneuron dendrites (t test; RP2: p = 0.0005; aCCi: p = 0.0194). Scale bars represent 10 μm. Dashed lines in (A) and (B) indicate midline. Anterior is up in (A) and left in (B).

Next, we investigated the role of postsynaptic motoneuron dendrites in determining connectivity. Previously, we showed that postsynaptic dendritic arbors regulate the number of inputs they receive by adjusting dendritic growth [28]. In motor networks, dendritic positioning has been suggested to be important in determining partner choice [29–33]. To investigate the role of dendritic arbor positioning in shaping connectivity, we changed the medio-lateral territories of motoneuron dendrites. Increasing dendritic sensitivity to the midline attractant Netrin, by targeted overexpression of the cognate receptor Frazzled/DCC, shifts RP2 dendrites from principally lateral to more medial neuropil regions. This shift leads to a reduction of laterally positioned dendrites, so that fewer are in proximity to the IN_lateral_ axon, and a concomitant increase of dendrites in the medial neuropil, which is innervated by another interneuron with a medial descending projection (IN_medial_) (Figure 5). As a result, the proportion of synapses between motoneurons and the IN_lateral_ is drastically reduced, whereas the proportion of synapses with the IN_medial_ is greatly increased, as compared to controls (Figure 5C; t test, p = 0.0005 and p = 0.0194 for RP2 and aCCi, respectively). Although these observations do not assay for changes in partner choice (RP2 and aCC receive connections from both IN_lateral_ and IN_medial_), these findings are compatible with a model where connections in motor systems emerge, to an extent, as a consequence of geographical overlap between presynaptic and postsynaptic terminals [30, 32].

In summary, our data point to the existence of mechanisms that allow postsynaptic neurons to determine in a cell-type-specific fashion the number of presynaptic synapses they accept. Clearly, geographical overlap between presynaptic and postsynaptic terminals is necessary for synaptic connections to form, and our experiments suggest that dendritic positioning mechanisms contribute to the emergence of connectivity.

## Discussion

There is currently no consensus among views on how patterns of connections develop in a motor network. On the one hand, a great deal of genetically encoded specificity is evident in parts of the mouse spinal cord. For example, group 1a afferents target motoneuron pools with accuracy, and their connectivity is buffered, so that normal information flow is largely maintained in the face of considerable disturbances [34]. Precision of wiring is perhaps most explicit in the selective positioning of inhibitory synapses by the so-called GABA pre-interneurons onto terminals of proprioceptive 1a sensory afferents. This precise and apparently invariant wiring is mediated by the expression of at least two sets of complementary heterophilic transsynaptic cell adhesion molecules [35, 36]. Contrasting with this view are studies from *Xenopus* tadpoles, where two-electrode recordings unequivocally demonstrated a surprising lack of specificity in synaptic connections during early stages of motor network development. Modeling based on these observations further suggests that such rather non-specific wiring patterns are able to generate swimming like motor outputs and that those patterns of connectivity could be formed simply through geographical overlap of coarsely defined presynaptic and postsynaptic termination zones [30, 32]. A limitation in those studies is that they look at groups of similar cells; this has precluded detailed insights at the level of individual synapses over developmental time. Here, we worked with identified partner neurons and studied how synaptic patterns in a motor network change, as the animal develops and grows.

### Connectivity Is Consolidated during Development

A striking observation from this study is that at the output face of the network, motoneurons increase synaptic contacts with existing presynaptic partners over time (Figures 1H and 1I). This correlates with previous observations that synaptic drive also increases during this period of larval development, although we do not yet have a physiological readout for the specific anatomical changes we detailed in this study. For motoneurons, the observed strengthening of existing connections is likely an adaptive mechanism that maintains the ability to effectively depolarize muscles as they enlarge during development [17]. Although we have not been able to assay for addition of new presynaptic partners during development, this wiring strategy contrasts with those proposed for cortical neurons, where pyramidal cells are thought to maximize the diversity of presynaptic inputs while keeping synapse number with each partner at a minimum [37].

### Connectivity in the Motor System Is Cell Specific yet Variable

Remarkably, reproducible cell-cell interactions during nervous system development can be genetically encoded, and this has been most clearly demonstrated with identified nerve cells of invertebrates—from highly specific substrate choices during axon path finding [38] to the selection of synaptic partners and the number of synapses formed [10, 39]. In the *Drosophila* larval motor system, we find that different motoneuron types have characteristic patterns of connections. For example, the likelihood of forming connections with the proprioceptive dda sensory neurons differs between the RP2 and aCC motoneurons (Figure 1I). Qualitative differences in the specificity of partner choice are also present in that the IN_lateral_ forms connections with motoneurons that innervate longitudinal body wall muscles (e.g., aCC, RP2, and RP3), but not with motoneurons thought to be antagonistic in operation, despite close proximity of their dendrites [24, 40] (Figure 2).

At the same time, this motor system also manifests a considerable degree of variability, both in the likelihood and the number of connections that form between motor and pre-motor interneurons. Although some connection patterns seem to become more reproducible during early phases of network maturation, such as those between the RP2 motoneuron and dda sensory terminals, by and large, our observations suggest that connectivity is inherently flexible and that it is the outcome of local cell-cell interactions, at least between most cells that we have been able to study. For example, two identical motoneurons (in different neuromeres) contacting the same IN_lateral_ axon can form quite different numbers of putative connections with the same presynaptic cell (Figure 4A). It is conceivable that these connections are variable because they are not critical to motor system operation, and it remains to be seen to what extent the observations of this study are representative of connectivity elsewhere in this network.

Where does the information that determines these connectivity outcomes reside? We found no correlation with segmental identity (Figure S4) or evidence for presynaptic patterning information: the number of presynaptic release sites that any one IN_lateral_ makes varies considerably, both between and within animals (left versus right homolog), and their distribution along the axon appears to be random, yet fairly even, with similar numbers of presynaptic sites per neuromere (Figures 4B and 4C). Most compatible with our data is the notion that patterns of connectivity are predominantly determined by the postsynaptic dendrites of motoneurons.

### Postsynaptic Terminals Regulate Synapse Number

We previously showed that motoneurons achieve a specific range of synaptic input by adjusting the growth of their dendritic arbors [28]. These structural adjustments mirror and complement homeostatic changes of neuronal excitable properties [41, 42]. Here, we show that different dendritic growth patterns lead to different connectivity outcomes. For example, aCC motoneurons are capable of initiating growth of dendritic branches from different parts of the cell, which can form connections with the ipsilateral and/or contralateral dda terminals, or neither (Figure 3). In an analogous situation, in the mouse retina, differences in dendritic growth lead to distinct connection patterns between different bipolar cells and presynaptic photoreceptor terminals [43]. We experimentally tested how dendritic positioning impacts connectivity. Changing the bias so that motoneurons preferentially elaborate their dendrites toward the ventral midline results in changes in connectivity, namely reductions in the proportion of synapses with the lateral IN_lateral_ and concomitant increases in connections with the medially located IN_medial_ axon (Figure 5). Although this experiment does not inform about partner choice, since both the IN_lateral_ and IN_medial_ are normally contacted by these motoneurons, it suggests that the number of connections is determined by the extent to which presynaptic and postsynaptic terminal arbors are targeted to common regions. These experiments in the *Drosophila* larva support observations and models on connectivity in the motor network of *Xenopus* tadpoles, which suggest that the connectivity matrix might be determined in considerable part by geographical overlap of coarsely defined presynaptic and postsynaptic territories [30, 32]. There is evidence that the conserved Slit-Robo and Netrin-Frazzled/DCC guidance cue systems define such territories for positioning axon tracts and regions of dendritic arborization in the CNS and that these can contribute to shaping synaptic connectivity [31, 33, 44–46]. That said, it remains to be established how the promiscuity of connections apparent in early *Xenopus* tadpoles changes over developmental time and to what extent hardwired specificity is genetically encoded elsewhere in the *Drosophila* or indeed in other motor networks.

## Experimental Procedures

The complete details of the experimental procedures are provided in the Supplemental Experimental Procedures.

### Fly Strains

Motoneurons were visualized using *RN2-Flp*^*A*^*,tub84B-FRT-stop-FRT-LexA.VP16, LexAOp-myr::Cerulean* [20]. Cholinergic pre-motor sensory neuron and interneuron Split-Gal4 expression lines, *BF29*^*VP16.AD*^ and *BF59*^*VP16.AD*^, were generated in a Split-Gal4-based enhancer trap screen [18]. Putative synapses were defined as sites where the presynaptic marker Brp::mRFP [21, 31] coincides with physical neuro-neuronal contact indicated by bimolecular fluorescence GRASP [22, 47] (Figures S1 and S3). Here, we use the full-length version of *UAS-brp::mRFP*. Its expression in IN_lateral_ axons is consistent with that of other synaptic markers (Figures S2A–S2C and S2G–S2K) and leads to similar numbers of Brp::XFP puncta as seen when expressing *UAS-brp-short::mStraw* (Figures S2D–S2F) [48].

### Dissections, Dye Fills, and Imaging

Nerve cords of staged larvae were dissected in Sørensen’s phosphate buffer (0.1 M [pH 7.2]), transferred onto a poly-L-lysine (Sigma) coated coverslip in saline, and imaged immediately with a Yokagawa CSU-22 confocal field scanner mounted on an Olympus BX51WI microscope, using a 63×/1.2 NA (Olympus) water immersion objective. Images were acquired at 0.3-μm z steps using a QuantEM cooled EMCCD camera (Photometrics), operated via MetaMorph software (Molecular Devices). Dye fills were carried out as detailed previously [31].

## Author Contributions

L.C., J.F.E., and M.L. designed the research. L.C., A.S.M., T.Y., S.D., J.F.E., and M.L. performed the research. L.C., A.S.M., J.F.E., and M.L. analyzed the data. L.C., A.S.M., J.F.E., and M.L. wrote the paper.

## Supplemental Information

Document S1. Supplemental Experimental Procedures and Figures S1–S5

Movie S1. Even Distribution of CD4::sp-GFP11 within Motoneuron Dendrites, Related to Figure 1 In a background of cholinergic neurons expressing the complementary fragment CD4::sp-GFP1-10 (*Cha7.4-Gal4; UAS-GFP1-10*), the whole dendritic tree of these CD4::sp-GFP11-expressing RP2 neurons displays reconstituted GFP fluorescence (green). Other examples are shown in Figure S3. Magenta indicates RP2 motoneurons as visualized with *w- ; LexAOp-CD4::spGFP11 ; RN2-FlpA, tub84B-FRT-stop-FRT-LexA.VP16, LexAOp-myr::mCherry*. Arrowheads point to the medio-lateral location of the IN_lateral_ (not visualized here).

Document S2. Article plus Supplemental Information
