# Supplementary material for: Development of Connectivity in a Motoneuronal Network in Drosophila Larvae
Source: Curr Biol. 2015 Mar 2;25(5):568–76. doi: 10.1016/j.cub.2014.12.056 (PMC4353686; doi:10.1016/j.cub.2014.12.056)
Supplement: Document S1. Supplemental Experimental Procedures and Figures S1–S5 [file mmc1.pdf]

Current Biology
Supplemental Information

Development of Connectivity in a Motoneuronal Network in *Drosophila* Larvae

Louise Couton, Alex S. Mauss, Temur Yunusov, Soeren Diegelmann, Jan Felix Evers, and Matthias Landgraf

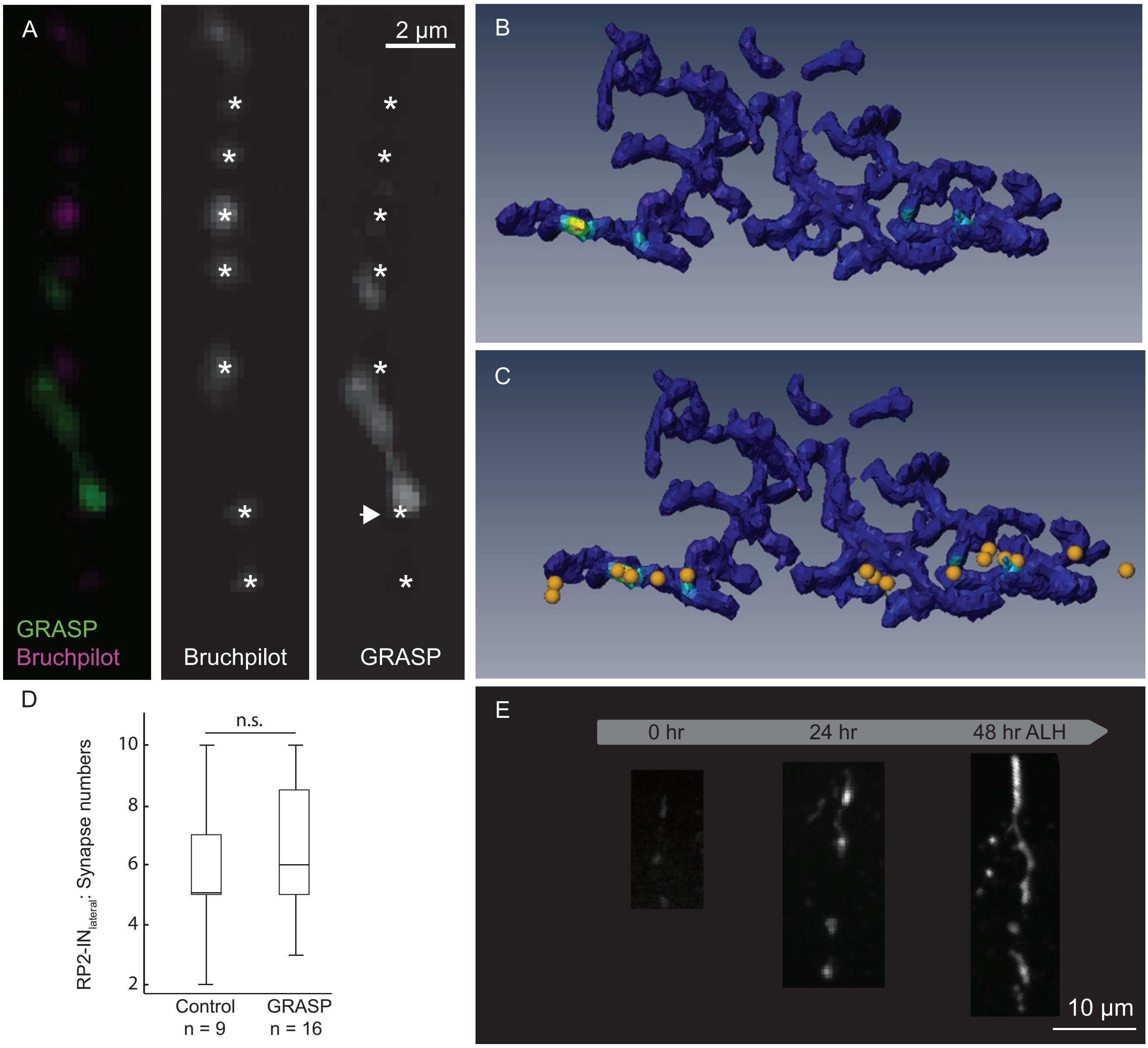

A
2 µm
GRASP
Bruchpilot
Bruchpilot
GRASP
B
C
D
n.s.
RP2-IN$_{lateral}$: Synapse numbers
10
8
6
4
2
Control
n = 9
GRASP
n = 16
E
0 hr
24 hr
48 hr ALH
10 µm

Figure S1

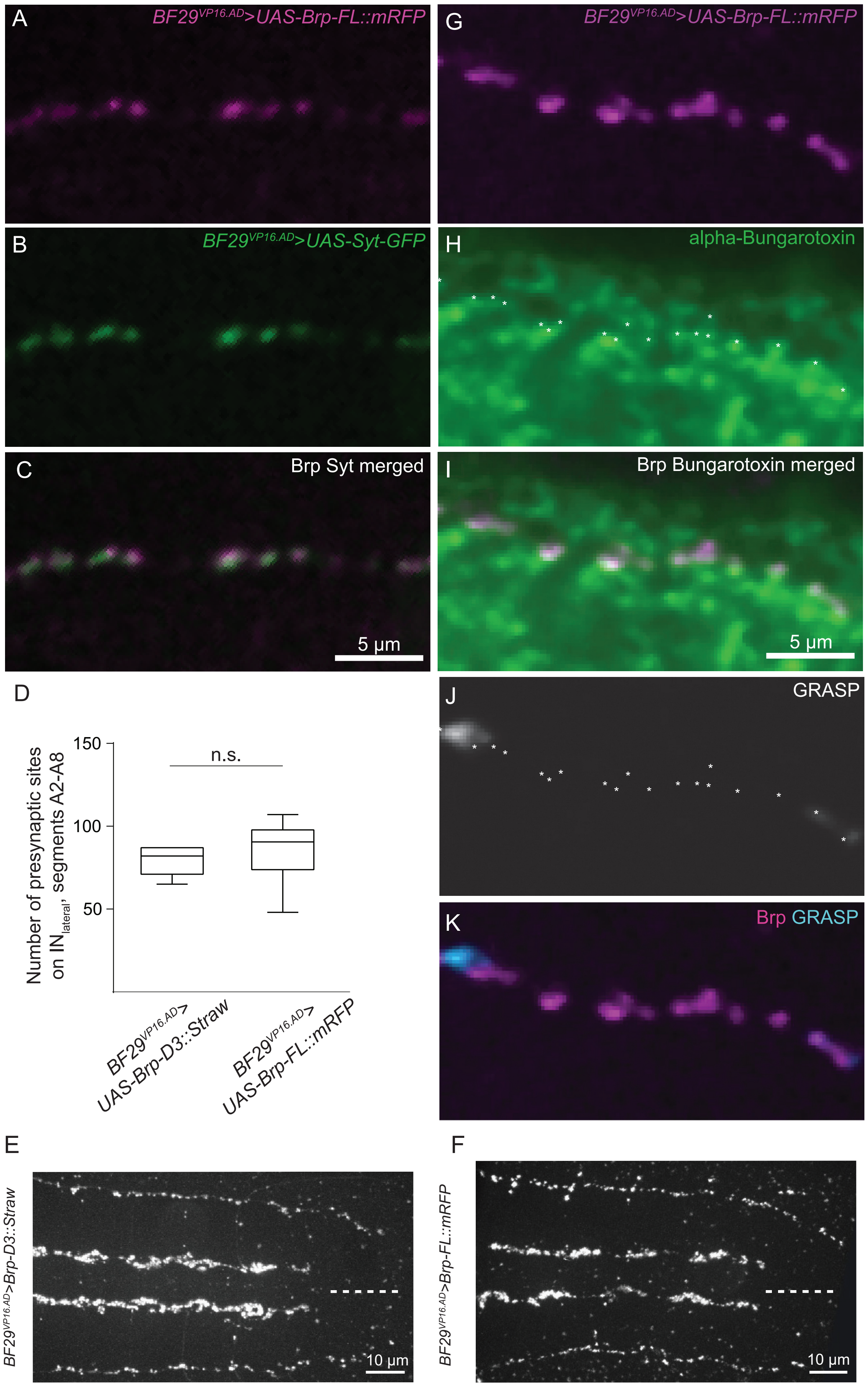
A
BF29VP16.AD>UAS-Brp-FL::mRFP
B
BF29VP16.AD>UAS-Syt-GFP
C
Brp Syt merged
5 µm
D
n.s.
150
100
50
Number of presynaptic sites on INlateral, segments A2-A8
BF29VP16.AD> UAS-Brp-D3::Straw
BF29VP16.AD> UAS-Brp-FL::mRFP
G
BF29VP16.AD>UAS-Brp-FL::mRFP
H
alpha-Bungarotoxin
I
Brp Bungarotoxin merged
5 µm
J
GRASP
K
Brp GRASP
E
BF29VP16.AD>Brp-D3::Straw
10 µm
F
BF29VP16.AD>Brp-FL::mRFP
10 µm

Figure S2

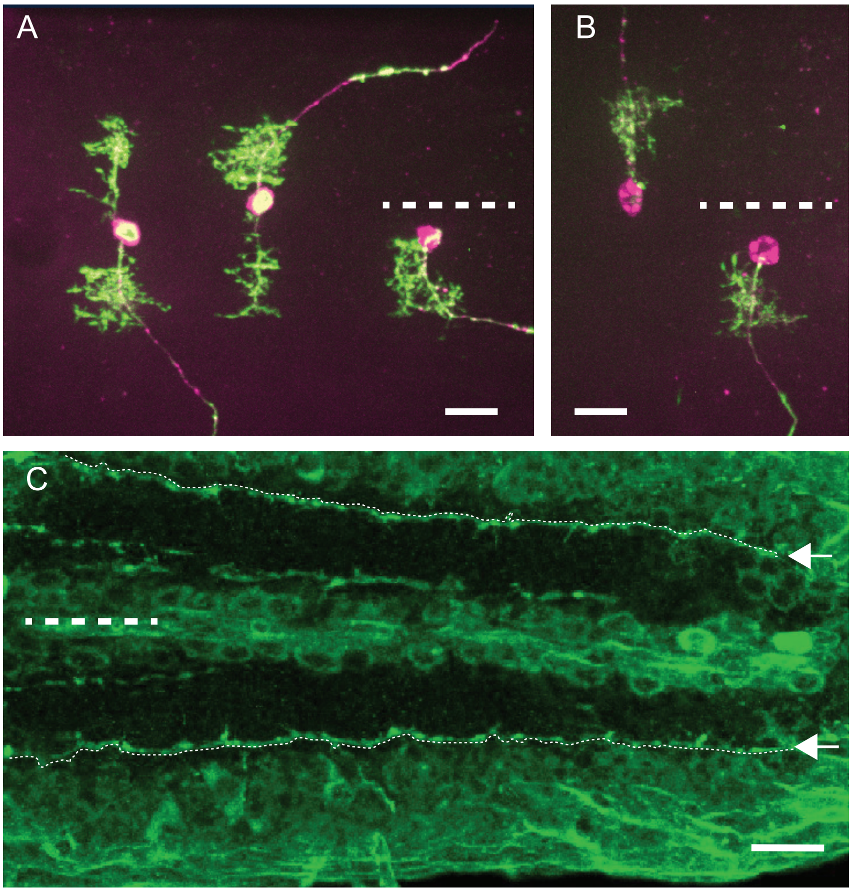

Figure S3

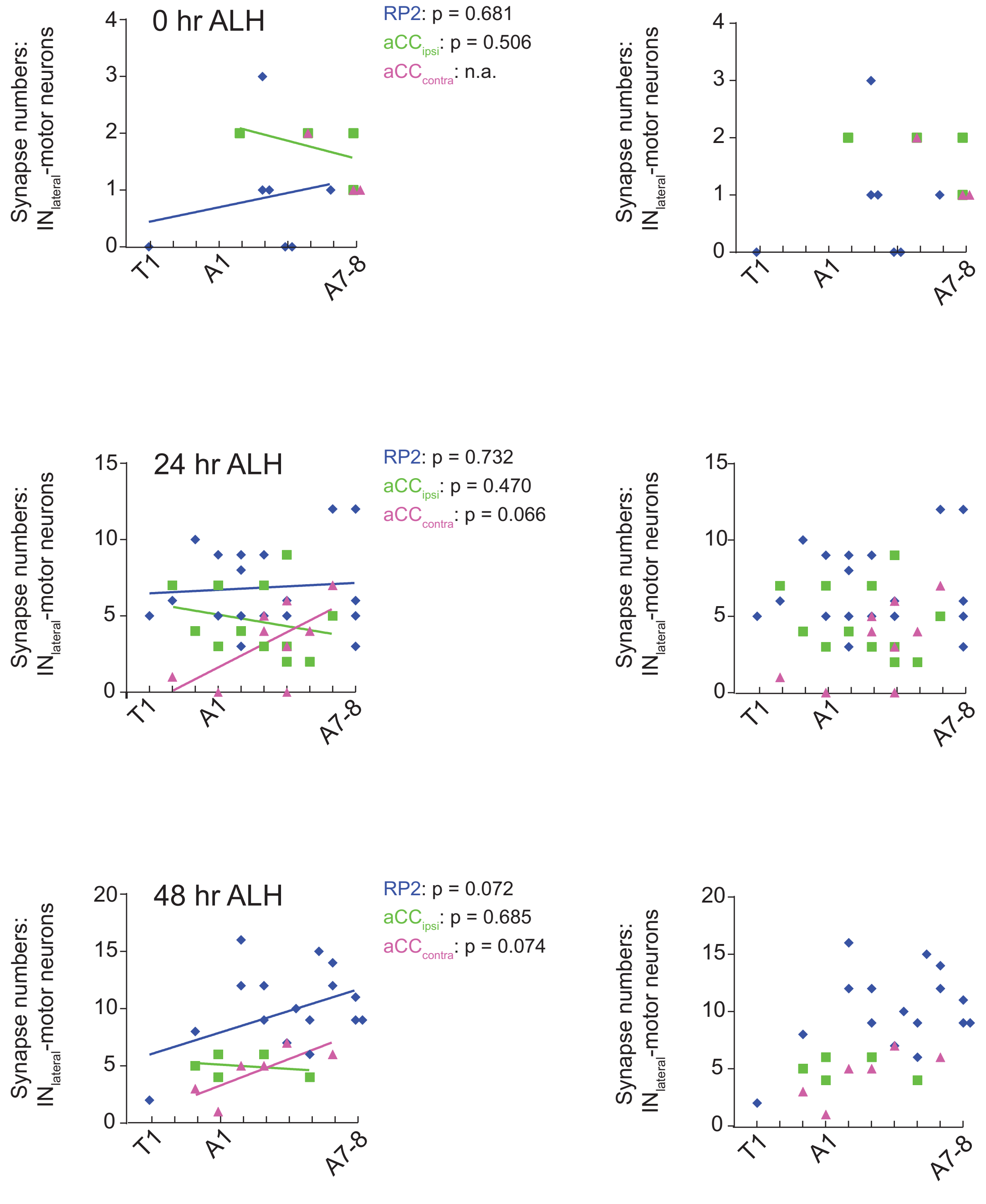
0 hr ALH
RP2: p = 0.681
aCC$_{ipsi}$: p = 0.506
aCC$_{contra}$: n.a.
24 hr ALH
RP2: p = 0.732
aCC$_{ipsi}$: p = 0.470
aCC$_{contra}$: p = 0.066
48 hr ALH
RP2: p = 0.072
aCC$_{ipsi}$: p = 0.685
aCC$_{contra}$: p = 0.074
Synapse numbers: IN$_{lateral}$-motor neurons
T1
A1
A7-8

Figure S4

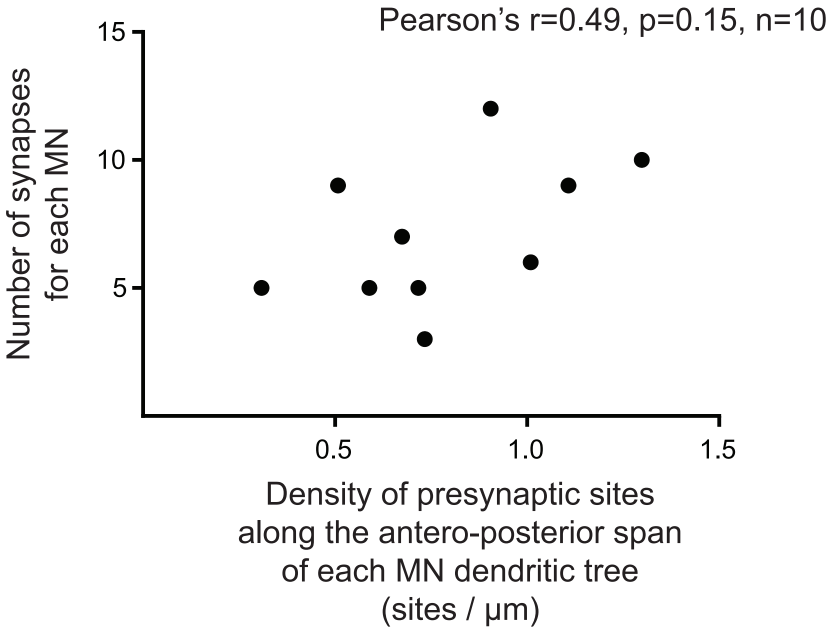
Pearson's r=0.49, p=0.15, n=10
15
10
5
Number of synapses for each MN
0.5
1.0
1.5
Density of presynaptic sites along the antero-posterior span of each MN dendritic tree (sites / µm)

Figure S5

Figure S1 (associated with Figure 1): GFP reconstitution does not manifestly bias synaptogenesis.

(A) Representative example showing GRASP is not systematically associated with synapses. In spite of an extensive GRASP signal (green), overlap with presynaptic Brp::mRFP puncta (magenta, asterisks) is only found at one site (arrowhead). (B and C) Partially reconstructed motoneuron dendritic tree with (B) and without (C) putative presynaptic sites shown (spheres). Regions where dendrites are closely apposed to presynaptic sites (<300 nm) are highlighted in cyan/yellow. Often dendritic regions are closely apposed to more than one presynaptic site. (D) Comparable numbers of putative synaptic contacts form between RP2 motoneuron dendrites and $IN_{lateral}$ axons with and without GRASP (t-test, p>0.05). Whiskers indicate the highest and lowest value of each dataset. In control experiments one of the GRASP components was omitted and RP2-$IN_{lateral}$ appositions were visualised with the genotype *w-;LexAOp-CD4::spGFP $_{11}$ /+;BF29$^{VP16.AD}$, Cha(7.4kb)$^{Gal4.DBD\ J8A1}$, UAS-brp::mRFP/RN2-Flp^{A}, tub84B-FRT-stop-FRT-LexA.VP16, 13xLexAOp2-IVS- myr::GFP*. RP2 dendritic trees were digitally reconstructed and appositions to $IN_{lateral}$ presynaptic sites within a 300nm radius were determined. (E) Motoneuron dendrites tend to progressively fasciculate with $IN_{lateral}$ axons as larvae grow older, producing a stripe-like GRASP signal along the $IN_{lateral}$ axon. Developmental increases in synapse numbers do not parallel the extent of this increase in the motoneuron dendrite-INlateral axon contact area. Anterior is up in A and E, left in B and C

Figure S2 (associated with Figure 1): Brp::mRFP localises consistently to other pre- and postsynaptic proteins and reports statistically similar numbers of presynaptic sites in the $IN_{lateral}$, compared to *UAS-brp-shortD3-Straw*

(A-C) The products of *UAS-Brp::mRFP* (A) and *UAS-Synaptotagmin::GFP* expressed within the same $IN_{lateral}$ neuron (B) co-localise consistently along the axon (C), suggesting Brp::mRFP is a faithful reporter of presynaptic sites. (D) Box plots depicting the numbers of presynaptic sites reported by each Brp construct in segments A2 to A8 of the $IN_{lateral}$ axon: (*brp-short$^{D3-Straw}$*; n = 6 neurons; *full length-brp::mRFP*; n = 18 neurons; n.s.: not significant, t-test: p>0.05). Whiskers indicate the highest and lowest value of each dataset. (E,F) Dorsal projections of confocal image stacks showing the patterns of expression of *BF29$^{VP16.AD}$*, *Cha(7.4kb)$^{Gal4.DBD\ J8A1}$* driving *UAS-brp-short$^{D3-Straw}$* (E) and *UAS-brp::mRFP* (full length) (F). (G-K) Brp::mRFP puncta along the $IN_{lateral}$ (G) and α-bungarotoxin-Alexa Fluor 647 puncta (H) are consistently apposed (I), supporting the idea that the presynaptic Brp::mRFP puncta are matched with postsynaptic acetylcholine receptor clusters. This is also true of GRASP-positive Brp puncta (J,K). Asterisks mark the locations of Brp::mRFP puncta in the α-bungarotoxin and GRASP channels.

Figure S3 (associated with Figure 1): Both split-GFP fragments distribute evenly in pre- and postsynaptic terminals.

(A, B) Within motoneuron dendrites, CD4::sp-GFP_{11} distributes evenly, as shown by the fact that in a background of cholinergic neurons expressing the complementary fragment CD4::sp-$GFP_{1\text{-}10}$ *(Cha7.4-Gal4; UAS-GFP $_{1\text{-}10}$)* [S1], the whole dendritic tree displays reconstituted GFP fluorescence (green) indicative of cell-cell contact with processes of cholinergic neurons, which provide the majority of synaptic input to motoneurons and constitute the vast majority of cells in the nerve cord. Magenta: RP2 and aCC motoneurons as visualised with *w- ; LexAOp-CD4::spGFP $_{11}$; RN2-Flp^{A}, tub84B-FRT-stop- FRT-LexA.VP16, LexAOp-myr::mCherry*. Confocal image stacks are shown as dorsal maximal z- projections, (but see also the movie corresponding to stack shown as panel B: **Movie S1**).

(C) Within the $IN_{lateral}$, CD4::sp-$GFP_{1\text{-}10}$ distributes evenly along the axon (arrows) as visualised with anti-GFP staining (Chicken anti-GFP, Abcam, dilution 1:10000), medially to the thin dotted outline on this panel. Unspecific background staining is seen in this instance on the surface of other cells along the midline and the lateral cortex. Genotype: *w-;+;$BF29^{VP16.AD}$, Cha(7.4kb)$^{Gal4.DBD\ J8A1}$, UAS-Brp::mRFP/UAS- GFP $_{1\text{-}10}$*. Scalebars: 10 µm, dashed lines: midline. Anterior is left.

Figure S4 (associated with Figure 4): The number of motoneuron-$IN_{lateral}$ synapses does not correlate with motoneuron segmental identity.

(A-C) Number of motoneuron-$IN_{lateral}$ synapses plotted against motoneuron segmental location, at 0 hr, 24 hr and 48 hr after larval hatching. Data are shown with (A-C) or without (A'-C') their linear regression lines. None of the regression lines differ significantly from a null slope, indicating that along the antero-posterior axis there are no significant segmental differences in the number of putative synapses formed between the RP2 and aCC motoneurons and the $IN_{lateral}$ interneuron.

Figure S5 (associated with Figure 4): The number of synapses within individual pairs of RP2-$IN_{lateral}$ does not correlate significantly with the local density of presynaptic sites.

The local density of presynaptic sites was calculated as the number of presynaptic sites/ µm along the stretch of $IN_{lateral}$ axon between the anterior-most and posterior-most branches of an RP2 dendritic tree. For each interneuron-motoneuron pair, this was plotted against the actual number of putative synapses, as reported by co-localisation of Brp::mRFP and cell-cell contacts -the latter indicated by reconstituted-GFP-. Data shown were collected 24 hr ALH.

Movie S1 (associated with Figure 1): Even distribution of CD4::sp-GFP$_{11}$ within motoneuron dendrites.

In a background of cholinergic neurons expressing the complementary fragment CD4::sp-GFP$_{1-10}$ (*Cha7.4-Gal4; UAS-GFP$_{1-10}$*) [S1], the whole dendritic tree of these CD4::sp-GFP$_{11}$-expressing RP2 neurons displays reconstituted GFP fluorescence (green). Other examples are shown in **Figure S3**. Magenta: RP2 motoneurons as visualised with *w- ; LexAOp-CD4::sp-GFP$_{11}$; RN2-FlpA, tub84B-FRT-stop-FRT-LexA.VP16, LexAOp-myr::mCherry*. Arrowheads point to the medio-lateral location of the IN$_{lateral}$ (not visualised here).

Supplemental experimental procedures

Staging larvae:

Flies were reared at 25°C on apple juice agar plates supplemented with yeast paste. Eggs (0-8 hr after egg laying) were incubated 16 hr at 29°C for optimal FLP recombinase activity. Stocks and recombinant chromosomes were generated using standard procedures. Transgenic lines were generated by BestGene Inc (California). Data were collected at three time points: 0 hr (first instar), 24 hr (second instar) and 48 hr ALH (third instar). For the first time point, newly hatched larvae were collected within 1 hour of hatching and used immediately. For the second and third time points, larvae were collected within 2 hours of hatching and transferred to fresh food until dissection, 24 hr or 48 hr later.

Visualisation of motoneurons and putative synaptic connections genotype:

w- ; LexAOp-CD4::sp-GFP$_{11}$/ + ; BF29$^{VP16.AD}$, Cha(7.4kb)$^{Gal4.DBD\ J8A1}$, UAS-brp::mRFP / RN2-Flp^{A}, tub84B-FRT-stop-FRT-LexA.VP16, LexAOp-myr::Cerulean, UAS-CD4::spGFP$_{1-10}$

Split-Gal4 expression pattern and distribution of presynaptic sites analysis genotype:

A balanced line carrying the *BF29$^{VP16.AD}$* or *BF59$^{VP16.AD}$* insertion was crossed to either *w- ; + ; Cha(7.4kb)$^{Gal4.DBD\ J8A1}$, UAS-EGFP* or *w- ; UAS-pmVenus/CyO ; Cha(7.4kb)$^{Gal4.DBD\ J8A1}$, UAS-brp::mRFP (full length)*

Dye fills

Freshly hatched larvae were dissected with ventral nerve cord and muscle field kept intact, as described by Baines and Bate [S2], then fixed with 3.7% formaldehyde for 10 min. Interneurons

identified by BF59-driven GFP expression, were impaled with sharp microelectrodes with 2mg/ml Neuro-DiO (Biotium) in EthOH and filled by delivering a hyperpolarizing current via an iontophoretic Dye Marker (Digitimer). Motor neurons were retrogradely labelled with DiD (Life Technologies) or Neuro-DiO (Biotium), dissolved in vegetable oil at 2mg/ml, as described by Landgraf, Bossing et al. [S3].

Image analysis

Image stacks were analysed using Amira software (FEI Visualization Sciences Group, France). To map the distribution of presynaptic sites along interneuron axons we digitally demarcated segments based on the pattern of sensory axon projections and marked Brp::mRFP puncta in segments A2 to A8, using the Amira landmark tool. We then exported the landmark coordinates to Matlab (The MathWorks, Natick, USA), normalised and analysed these using custom made functions. To assess the number of putative synapses between motor neurons and their presynaptic partners, three channel image stacks showing myr::Cerulean (postsynaptic motor neuron), Brp::mRFP (presynaptic sites) and GRASP GFP cell-cell contact were loaded into Amira software. We marked the locations of all Brp::mRFP puncta and recorded those co-localising with GRASP signal as putative synapses. To assess synapse numbers, we only considered motor neurons located in segments A2 to A6, which are more similar in function as compared to homologues located elsewhere in the nerve cord. However, when addressing the effect of segmental identity (Figure S4), we used a wider range of motoneurons from T1 to A8. Reconstructions of dendritic trees and calculation of proximity to the presynaptic partners were performed using a plugin for Amira developed by J-F Evers [S4, 5].

Statistics

Data were plotted and analyzed using Matlab (The MathWorks, Natick, USA) or Prism software (GraphPad Software). At the $IN_{lateral}$-motoneuron interface (**Figure 1H**), changes in synapse numbers through time were tested for using ANOVA and a post-test for trend. At each time point, differences in synapse number between different types of dendritic arbors were tested for using ANOVA and the uncorrected Fisher's LSD test. At the sensory-motor interface (**Figure 1I**), changes in the proportion of motoneurons making synaptic contact were tested for using a Chi-square test for trend. At each time-point, the proportions of each motoneuron type making contact with the sensory partner were compared using Fisher's exact test. In order to assess whether the presynaptic site distribution differed from random (**Figure 4B and C**), we used Matlab to create a set of 17 virtual axons with the same number of presynaptic sites as our actual sample, though

randomly distributed. We then compared both distributions using the Kolmogorov-Smirnov two sample test. In order to ask whether the distribution of synaptic contacts between $IN_{lateral}$ and IN_{medial} changes when the dendritic trees are shifted towards the midline (relates to **Figure 5C and D**), we compared the proportion of synapses located on the $IN_{lateral}$ in the control and frazzled-expressing groups: t-tests were applied to RP2, $aCC_{ipsilateral}$ and $aCC_{contralateral}$ For each of these dendritic trees, we only considered the set of interneuron axons located in the same hemisegment, since the change of dendritic territory is not such that these trees could make connections in the contralateral hemisegment. We used t-tests to compare the average numbers of synapses reported with and without GRASP (**Figure S1**), as well as to compare the numbers of presynaptic sites reported by *UAS-Brp-Straw-D3* and *UAS-Brp::mRFP* (**Figure S2**). We applied a regression analysis to check whether the changes in synapse numbers follow an antero-posterior trend (**Figure S4**). Finally, we used Pearson's R to ask about a possible correlation between the local density of presynaptic sites and the actual number of synapses (**Figure S5**).

Immunocytochemistry

The location of interneurons in *$BF29_{VP16.AD}$* was determined by staining nerve cords against Fasciclin II (mouse anti-Fasciclin II 1D4, 1:20, DSHB) [S6] and Sex comb reduced (mouse anti-Scr 6H4.1, 1:100, DSHB) [S7]. Cholinergic neurotransmitter identity of interneurons in the *BF29VP16.AD* and a *BF59VP16.AD* expression lines were confirmed by staining for mouse anti-ChaT 4B1 (1:100, DSHB) [S8]. Absence of staining for other major transmitters was confirmed by staining with anti-GABA (rabbit anti-GABA 1:1,000 dilution, Sigma) [S9] and anti-DvGlut (rabbit anti-DvGlut 1:500) [S10]. Secondary antibodies were donkey anti-rabbit Cy3 (1:400, Jackson ImmunoResearch), donkey anti-mouse Cy5 (1:400, Jackson ImmunoResearch), donkey anti-mouse CF568 (1:600, Biotium). To label cholinergic receptors, fixed preparations with dye-labeled neurons were incubated with alpha-Bungarotoxin-A488 (2µM) in saline for a few hours. Alternatively, when testing whether Brp::mRFP puncta localised consistently with (postsynaptic) cholinergic receptors, a 2µM solution of alpha-Bungarotoxin-A647 (Life Technologies) was applied for 10 minutes on freshly dissected, unfixed nerve cords expressing Brp::mRFP, rinsed twice for 2 minutes in physiological saline and imaged immediately.

Molecular biology

Mapping the $BF29^{VP16.AD}$ insertion: The genomic insertion site of *$ET^{VP16.AD}$* in the *$BF29^{VP16.AD}$* enhancer-trap line was determined by inverse PCR and mapped to the *eyegone* locus, using the protocol published on the BDGP website. Briefly, genomic DNA isolated from a balanced stock

that only carried the $BF29^{VP16.AD}$ insertion was digested with Sau3A and MspI (New England Laboratories). Fragments were then self-ligated and amplified by inverse PCR using primers Pry4 and Plw3-1.

Construction of LexAOp-myr::Cerulean: The coding sequence for the fluorophore Cerulean and the myristoylation sequence were PCR amplified using primers suitable for multisite Gateway cloning (Life Technologies) to create pEntry vectors (P1-myr-P5r, P5-Cerulean-P2). These vectors were combined in a two fragment recombination reaction into the pLOT-W vector [S11] to fuse the myr::Cerulean downstream of the LexA operon. DNA was purified using a Qiagen Midi Kit and transgenic lines were generated by BestGene Inc. (Chino Hills, CA, USA).

Construction of 13xLexAOp2-frazzled-myc: cDNA coding for *fraB-6xMyc* [S12] was PCR amplified and via Infusion (Clontech) cloned into a XhoI –XbaI linearised pJFRC19 vector backbone [S13] to generate *p13xLexAOp2-IVS-frazzled-myc*, subsequently integrated via PhiC31-mediated recombination into the Su(Hw)attP6 landing site (2L, cytogenetic map: 24D) (BestGene, California) [S14].

Control for the validity of the GRASP approach

A potential concern with using the GRASP reporter is that the GFP reconstitution might lead to strong cell-cell adhesion, which in turn may lead to artefactual synapse formation between apposed cells. To test this, we carried out control experiments, in which one of the GRASP components was omitted and then imaged the RP2-$IN_{lateral}$ contacts, using the following genotype:

w-;LexAOp-CD4:spGF$_{11}$ /+ ;BF29$^{VP16.AD}$, Cha(7.4kb)$^{Gal4.DBD\ J8A1}$, UAS-brp::mRFP/RN2-Flp^{A}, tub84B-FRT- stop-FRT-LexA.VP16, 13xLexAOp2-IVS-myr::GFP

We then digitally reconstructed RP2 dendritic trees and identified the $IN_{lateral}$ presynaptic sites closely apposed to RP2 dendrites, within a 300 nm radius. Statistically comparable numbers of presynaptic sites form between RP2 motor and $IN_{lateral}$ interneurons with and without presence of bimolecular fluorescence complementation (t-test, $p>0.05$), suggesting that, at least in this experimental system, GRASP mediated cell-cell adhesion does not obviously lead to the formation and/or maintenance of supernumerary synapses (**Figure S1**). A second argument supporting the validity of the GRASP approach in this study is that motoneuron dendrites tend to

increasingly fasciculate with $IN_{lateral}$ axons as larvae grow older. As a result, the GRASP signal changes from an initially punctate appearance at the first larval instar stage to a linear pattern by the third larval instar. This dramatic increase in cell-cell apposition between partner neurons is not paralleled by steep increases in synapse number; *i.e.* large areas of GRASP signal exist that show no overlap with presynaptic sites. These observations suggest that while the bimolecular fluorescence complementation of the GRASP method may mediate artefactual cell-cell adhesion, this appears to be synaptogenically inert.

Supplemental references

S1. Salvaterra, P.M., and Kitamoto, T. (2001). *Drosophila* cholinergic neurons and processes visualized with Gal4/UAS-GFP. Brain Res Gene Expr Patterns *1*, 73-82.

S2. Baines, R.A., and Bate, M. (1998). Electrophysiological development of central neurons in the *Drosophila* embryo. J Neurosci *18*, 4673-4683.

S3. Landgraf, M., Bossing, T., Technau, G.M., and Bate, M. (1997). The origin, location, and projections of the embryonic abdominal motorneurons of *Drosophila*. J Neurosci *17*, 9642-9655.

S4. Schmitt, S., Evers, J.F., Duch, C., Scholz, M., and Obermayer, K. (2004). New methods for the computer-assisted 3-D reconstruction of neurons from confocal image stacks. Neuroimage *23*, 1283-1298.

S5. Evers, J.F., Schmitt, S., Sibila, M., and Duch, C. (2005). Progress in functional neuroanatomy: precise automatic geometric reconstruction of neuronal morphology from confocal image stacks. J Neurophysiol *93*, 2331-2342.

S6. Vactor, D.V., Sink, H., Fambrough, D., Tsoo, R., and Goodman, C.S. (1993). Genes that control neuromuscular specificity in *Drosophila*. Cell *73*, 1137-1153.

S7. Glicksman, M.A., and Brower, D.L. (1988). Expression of the Sex combs reduced protein in *Drosophila* larvae. Dev Biol *127*, 113-118.

S8. Takagawa, K., and Salvaterra, P. (1996). Analysis of choline acetyltransferase protein in temperature sensitive mutant flies using newly generated monoclonal antibody. Neuroscience Research *24*, 237-243.

S9. Wilson, R.I., and Laurent, G. (2005). Role of GABAergic inhibition in shaping odor-evoked spatiotemporal patterns in the *Drosophil*a antennal lobe. J Neurosci *25*, 9069-9079.

S10. Mahr, A., and Aberle, H. (2006). The expression pattern of the Drosophila vesicular glutamate transporter: a marker protein for motoneurons and glutamatergic centers in the brain. Gene Expr Patterns *6*, 299-309.

S11. Diegelmann, S., Bate, M., and Landgraf, M. (2008). Gateway cloning vectors for the LexA-based binary expression system in *Drosophila*. Fly (Austin) *2*.

S12. Bashaw, G.J., and Goodman, C.S. (1999). Chimeric axon guidance receptors: the cytoplasmic domains of slit and netrin receptors specify attraction versus repulsion. Cell *97*, 917-926.

S13. Pfeiffer, B.D., Ngo, T.T., Hibbard, K.L., Murphy, C., Jenett, A., Truman, J.W., and Rubin, G.M. (2010). Refinement of tools for targeted gene expression in *Drosophila*. Genetics *186*, 735-755.

S14. Ni, J.Q., Liu, L.P., Binari, R., Hardy, R., Shim, H.S., Cavallaro, A., Booker, M., Pfeiffer, B.D., Markstein, M., Wang, H., et al. (2009). A Drosophila resource of transgenic RNAi lines for neurogenetics. Genetics 182, 1089-1100.
