# Supplementary figures and images for: Development of Connectivity in a Motoneuronal Network in Drosophila Larvae
Source: Curr Biol. 2015 Mar 2;25(5):568–76. doi: 10.1016/j.cub.2014.12.056 (PMC4353686; doi:10.1016/j.cub.2014.12.056)

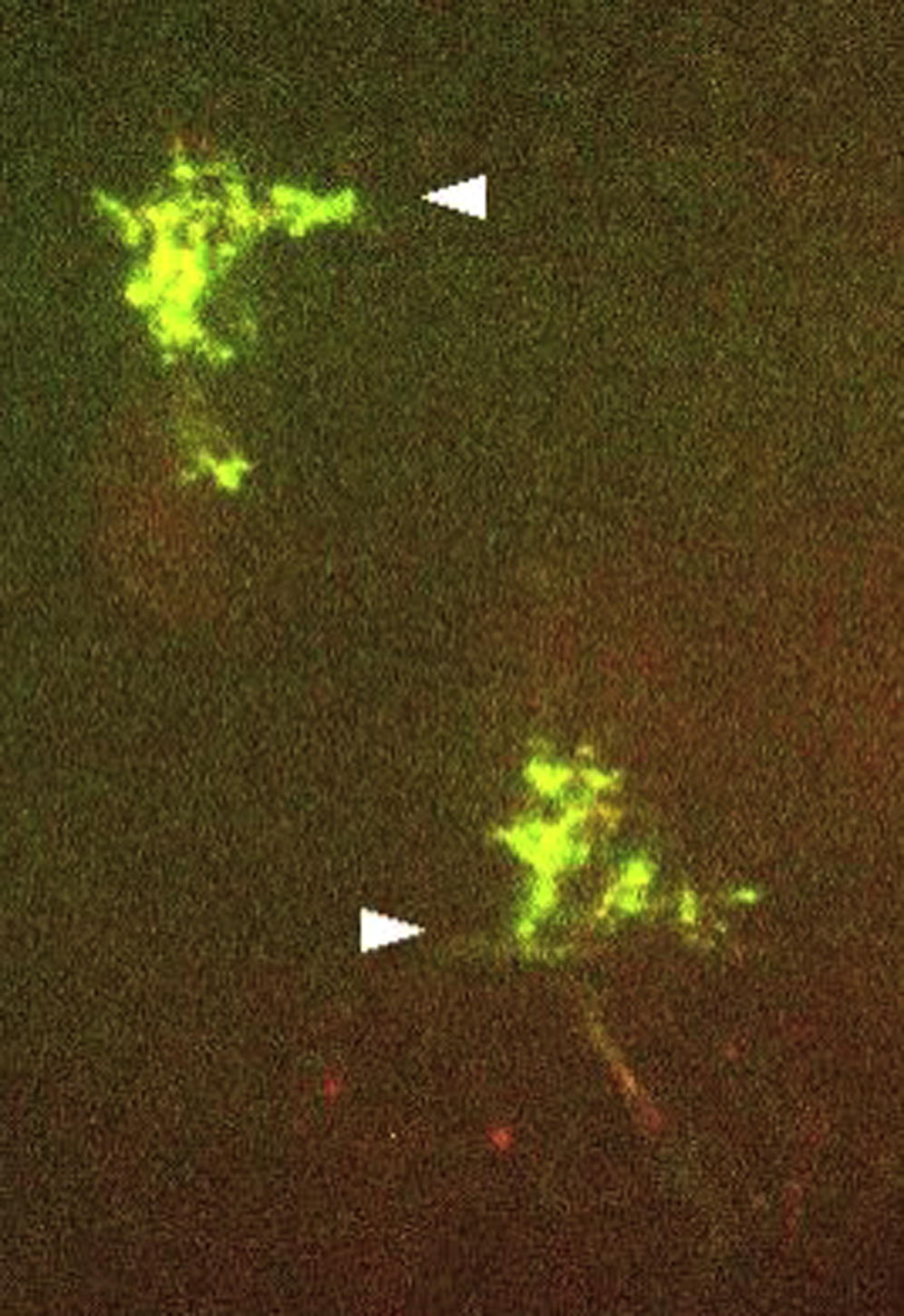

Supplement: Movie S1. Even Distribution of CD4::sp-GFP11 within Motoneuron Dendrites, Related to Figure 1 — In a background of cholinergic neurons expressing the complementary fragment CD4::sp-GFP1-10 (Cha7.4-Gal4; UAS-GFP1-10), the whole dendritic tree of these CD4::sp-GFP11-expressing RP2 neurons displays reconstituted GFP fluorescence (green). Other examples are shown in Figure S3. Magenta indicates RP2 motoneurons as visualized with w- ; LexAOp-CD4::spGFP11 ; RN2-FlpA, tub84B-FRT-stop-FRT-LexA.VP16, LexAOp-myr::mCherry. Arrowheads point to the medio-lateral location of the INlateral (not visualized here). [file mmc2.jpg]
