# Supplementary material for: Development of Connectivity in a Motoneuronal Network in Drosophila Larvae
Source: Curr Biol. 2015 Mar 2;25(5):568–76. doi: 10.1016/j.cub.2014.12.056 (PMC4353686; doi:10.1016/j.cub.2014.12.056)
Supplement: Document S2. Article plus Supplemental Information [file mmc3.pdf]

Article

Current Biology

Development of Connectivity in a Motoneuronal Network in *Drosophila* Larvae

Highlights

- Growing motoneurons consolidate synapses with existing presynaptic partners
- Motoneuron dendritic arbors are active parties in setting connectivity patterns
- Cell-type-specific features coexist with variations at the individual cell level
- Motoneuron wiring strategies may contrast with those of sensory neurons

Authors

Louise Couton, Alex S. Mauss, ..., Jan Felix Evers, Matthias Landgraf

Correspondence

jan-felix.evers@cos.uni-heidelberg.de

In Brief

The development of neural connections has mainly been studied in the context of sensory networks. Couton et al. explore how, in a motor network, synaptic connections change during development, finding that connectivity patterns show considerable variability between cells and that motoneuron dendrites play an active role in shaping these.

Couton et al., 2015, Current Biology *25*, 568–576
March 2, 2015 ©2015 The Authors
http://dx.doi.org/10.1016/j.cub.2014.12.056

Current Biology 25, 568–576, March 2, 2015 ©2015 The Authors http://dx.doi.org/10.1016/j.cub.2014.12.056

Article

Development of Connectivity in a Motoneuronal Network in *Drosophila* Larvae

Louise Couton,[1] Alex S. Mauss,[1,3] Temur Yunusov,[1,4] Soeren Diegelmann,[1,5] Jan Felix Evers,[1,2,6,*] and Matthias Landgraf[1,6]
[1]Department of Zoology, University of Cambridge, Cambridge CB2 3EJ, UK
[2]Centre for Organismal Studies, Ruprecht-Karls-Universität, 69120 Heidelberg, Germany

Summary

Background: Much of our understanding of how neural networks develop is based on studies of sensory systems, revealing often highly stereotyped patterns of connections, particularly as these diverge from the presynaptic terminals of sensory neurons. We know considerably less about the wiring strategies of motor networks, where connections converge onto the dendrites of motoneurons. Here, we investigated patterns of synaptic connections between identified motoneurons with sensory neurons and interneurons in the motor network of the *Drosophila* larva and how these change as it develops.
Results: We find that as animals grow, motoneurons increase the number of synapses with existing presynaptic partners. Different motoneurons form characteristic cell-type-specific patterns of connections. At the same time, there is considerable variability in the number of synapses formed on motoneuron dendrites, which contrasts with the stereotypy reported for presynaptic terminals of sensory neurons. Where two motoneurons of the same cell type contact a common interneuron partner, each postsynaptic cell can arrive at a different connectivity outcome. Experimentally changing the positioning of motoneuron dendrites shows that the geography of dendritic arbors in relation to presynaptic partner terminals is an important determinant in shaping patterns of connectivity.
Conclusions: In the *Drosophila* larval motor network, the sets of connections that form between identified neurons manifest an unexpected level of variability. Synapse number and the likelihood of forming connections appear to be regulated on a cell-by-cell basis, determined primarily by the postsynaptic dendrites of motoneuron terminals.

Introduction

Much of our current view of how sets of synaptic connections form and change during nervous system development is derived from studies of sensory systems [1–6]. The connections that sensory neurons form are often tightly constrained, enabling the formation of accurate sensory maps, with numbers and distributions of synapses appropriate for network operation [7, 8]. Connectivity at lower-order synapses of the network can be almost invariant and cell autonomously specified. For example, *Drosophila* photoreceptor neurons reproducibly form ~50 synapses with specific postsynaptic lamina cells, irrespective of photoreceptor function or visual system defects [9, 10]. At higher-order synapses, in contrast, connectivity can be rather variable, reflecting both experience-dependent plasticity and distinct wiring strategies [11, 12]. For example, randomized connections in the mushroom body are thought to maximize coding space [13, 14].

Here, we focus on the much less well-explored development of connectivity within a motor network. Motor systems manifest a great deal of flexibility, including their ability to adjust to changes in muscle size with growth and exercise, thus maintaining the capacity to trigger effective muscle contractions. This has been most extensively studied at the neuromuscular junction where the growth of the presynaptic terminal is matched with that of the postsynaptic muscle, regulated by muscle-derived retrograde signals [15, 16]. In addition, motoneurons also adjust centrally through changes in the size and connectivity of their dendritic arbors [17].

To investigate patterns of connectivity in a motor network and how these change as the animal develops and grows, we used the *Drosophila* larva as a model. We developed a paradigm for studying identified partner neurons at the level of individual synaptic sites across different developmental stages. We asked the following questions: (1) How does connectivity change as the motor network develops? (2) How reproducible or variable are the sets of connections that form? (3) Is there evidence of synaptic patterning information residing with the presynaptic or postsynaptic partner? We show that from hatching to later larval stages, existing connections are progressively consolidated by addition of synapses. We find that while patterns of connections are specific to each motoneuron type, considerable variability remains. Moreover, connectivity appears to be set on a cell-by-cell basis by the dendritic arbors of motoneurons, and dendritic positioning is a determinant of the connections that motoneurons make. Together, these findings argue in favor of a flexible regulation of connectivity in the assembly of the larval crawling circuit.

Results

Imaging Putative Synaptic Connections between Identified Neurons in a Developing Motor System

To study the emergence of synaptic connectivity in a motor network as it develops, we generated genetic tools for reliably visualizing and manipulating identified, connecting neurons in the *Drosophila* larval nerve cord. For pre-motor partner neurons, we fractionated through an intersectional "split-Gal4" enhancer trap screen [18] the set of cholinergic interneurons and sensory neurons, which provide the synaptic drive to motoneurons in this system [19]. From >3,000 lines, we identified those with sparse expression and terminations in the motor neuropile. Single motoneurons ("aCC" and "RP2") were

[3]Present address: Max Planck Institute of Neurobiology, 82152 Martinsried, Germany
[4]Present address: Sainsbury Laboratory, University of Cambridge, Cambridge CB2 1LR, UK
[5]Present address: Department of Biology, Institute of Zoology, Chemin du Musée 10, 1700 Fribourg, Switzerland
[6]Co-senior author
*Correspondence: jan-felix.evers@cos.uni-heidelberg.de
This is an open access article under the CC BY license (http://creativecommons.org/licenses/by/4.0/).

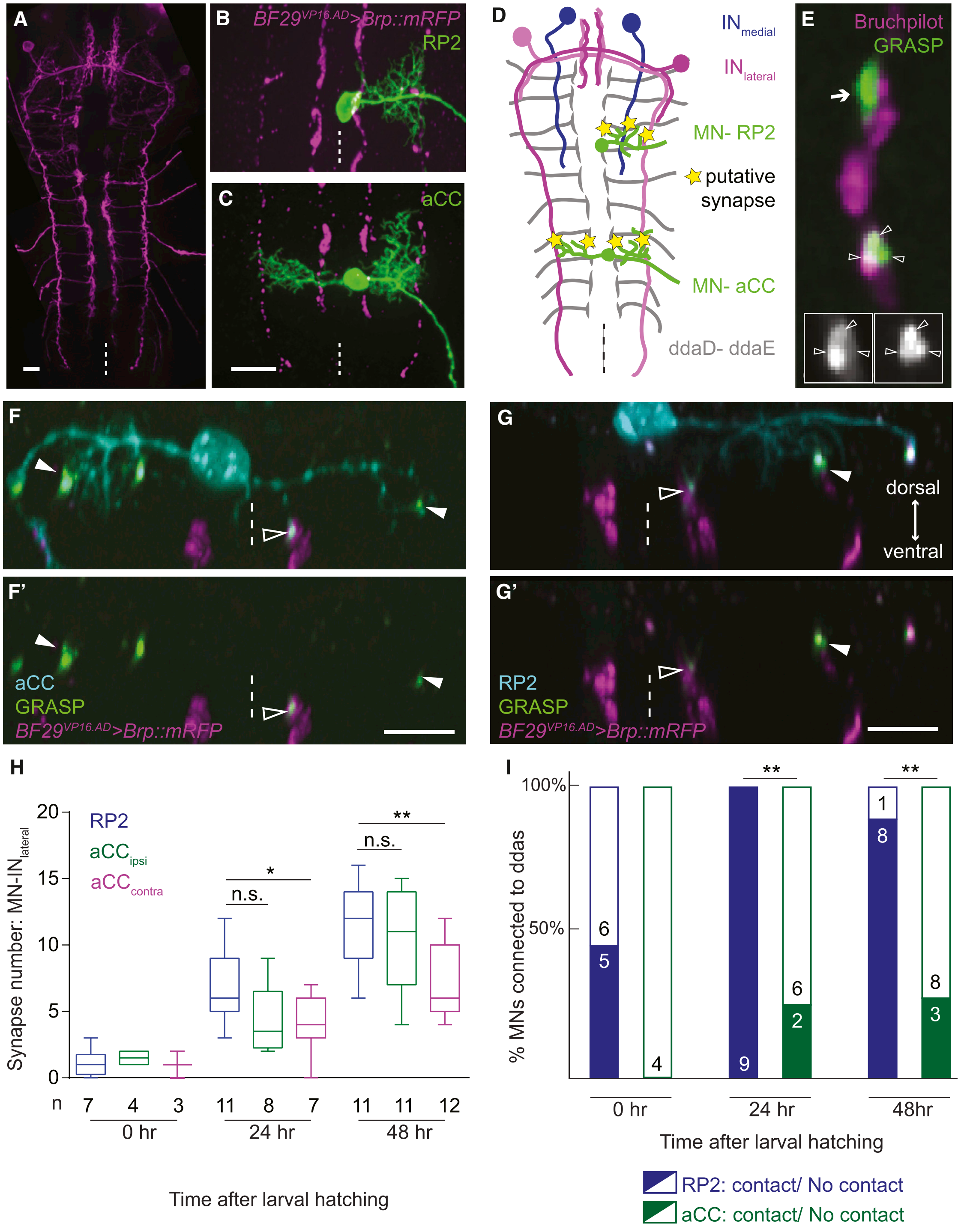

Figure 1. Genetic Tools to Differentially Label Partner Neurons Reveal a Motoneuron-Specific Pattern of Connectivity during Development

(A and D) Intersection of the split-Gal4 line *BF29*$^{VP16.AD}$ with *Cha(7.4kb)*$^{Gal4.DBD\ J8A1}$ targets Gal4 activity to two pairs of descending interneurons, namely a lateral ($IN_{lateral}$) and a medial (IN_{medial}) interneuron on each side of the midline, as well as to the segmentally repeated dda sensory neurons (ddaD and ddaE).

(B–D) Two motoneurons (aCC and RP2) that make connections with these interneurons are visualized with a LexA-Flpout system.

(E) Synaptic contacts between partner neurons (arrowheads) are identified by co-localization of the presynaptic active zone marker Bruchpilot (Brp) and the cell-cell contact reporter (GRASP). The arrow points to a contact region between partner neurons that is devoid of presynaptic Brp::mRFP and therefore not considered a putative synaptic site. Insets show the synapse area as separate channels.

(F–G′) Cross section views of nerve cords showing the location of aCC and RP2 motoneurons, relative to their presynaptic sites from their common partners, $IN_{lateral}$ (filled arrowheads) and dda terminals (open arrowheads). (F′) and (G′) show the GRASP and Brp channels separately.

(H and I) Quantification of the development of connections between the aCC and RP2 motoneurons and the $IN_{lateral}$ (H) and ddas (I) during larval life. Boxplots show the median of the distribution (middle line), the 75th (upper limit of box) and 25th (lower limit of box) percentile; whiskers indicate the highest and lowest value of each dataset. Each of the dendritic arbors shows a significant increase in connectivity with the $IN_{lateral}$ over time: ANOVA (p values < 0.01) with post tests for linear trend: $p < 0.0001$ for RP2 and aCC ipsilateral; $p = 0.0025$ for aCC contralateral arbor. RP2 arbors had a significantly different number of

(legend continued on next page)

visualized via a LexA/LexAOp and FLP recombinase-based quaternary system [20] (see Supplemental Experimental Procedures). To resolve synaptic sites, we combined the presynaptic active zone marker *UAS-brp::mRFP* [21] with the GFP reconstitution across synaptic partners (GRASP)-based reporter for cell-cell contacts [22]. Brp::mRFP-positive presynaptic specializations that coincide with physical appositions of presynaptic and postsynaptic membranes, as reported by GRASP, were scored as putative synapses (Figures 1A–1G′; see Figures S1–S3 and Movie S1 for technical validation). We thus charted patterns of connectivity during larval development, from 0 hr after larval hatching (ALH) to the third instar stage (48 hr ALH), between the aCC and RP2 motoneurons and some of their presynaptic partners, made accessible to analysis by the Split-Gal4 line $BF29^{VP16.AD}$: two intersegmental descending interneurons and the ddaD and ddaE proprioceptive sensory neurons [23] (Figures 1H and 1I) (see Supplemental Experimental Procedures for details).

We focused on the lateral interneuron ($IN_{lateral}$) within the $BF29^{VP16.AD}$ expression pattern; its axon descends contralaterally from the sub-esophageal ganglion to segment A8 and forms putative en passant synapses with intersegmental nerve motoneurons. In mid-abdominal segments (A2–A6), the number of putative synaptic connections between this $IN_{lateral}$ and the RP2 motoneuron increases steadily with developmental time from an average of 0.86 ± 0.26 at 0 hr ALH to 6.73 ± 0.78 at 24 hr ALH to 11.09 ± 0.97 at 48 hr ALH (n = 7–11) (Figure 1H). This developmental increase in synapse number is compatible with electrophysiological recordings from these motoneurons [17]. $IN_{lateral}$ axons also form putative synapses with the two dendritic sub-arbors of the aCC motoneuron. The larger ipsilateral arbor, located on the same side as the aCC soma, receives more putative synapses from the $IN_{lateral}$ than the smaller sub-arbor on the contralateral side (Figure 1H). Both RP2 and aCC project to dorsal body wall muscles. To extend these observations to motoneurons that innervate ventral muscles, we manually labeled RP3 motoneurons with the lipophilic tracer dye DiD and charted co-localization with $IN_{lateral}$ Brp::mRFP sites as putative connections. Here, too, we found that the number of putative connections between this pair of neurons increases with developmental time, from 1 synapse (±0, n = 3) at 0 hr ALH to an average of 3.6 synapses (±0.4, n = 5) at 24 hr ALH.

Next, we looked at cell-type-specific differences in connectivity. These are most evident in the likelihood with which the RP2 and aCC motoneurons receive putative synapses from the ddaD and ddaE sensory terminals (the high density of Brp::mRFP puncta in these sensory terminals prevents resolution of individual puncta; Figures 1F, 1G, and 1I). As larvae develop, this sensory-motor connection becomes increasingly frequent, although throughout aCC, motoneurons have a significantly lower probability than RP2 of forming putative synapses with these dda sensory terminals (Figure 1I). In addition, we found that motoneurons such as RP3, which are similar in operation to RP2 and aCC, i.e., in innervating longitudinal body wall muscles, also form putative synapses with the presynaptic $IN_{lateral}$, while motoneurons innervating antagonistic transverse muscles [24] do not, even though their dendrites arborize within reach of the $IN_{lateral}$ axon (Figures 2A–2B″). For another pre-motor interneuron, IN_{BF59}, labeled with the $BF59^{VP16.AD}$ expression line (Figure 2C), we resolved single cells by injecting IN_{BF59} interneurons expressing *UAS-brp::mRFP* with the lipophilic tracer dye, Neuro-DiO, and different motoneurons with the spectrally distinct DiD. Co-localization of these three markers (Neuro-DiO, Brp::mRFP, and DiD) was taken as indicative of a putative synapse (Figures 2C–2F). The data suggest that different motoneurons, projecting to dorsal (aCC, RP2), lateral (MN-LL1), and ventral (RP3) muscles, may have different likelihoods of contacting the IN_{BF59}.

In summary, in this motor network, the number of putative synapses between partner neurons generally increases as the network matures and the animal grows. Different motoneurons have different likelihoods of forming synapses with the same sets of presynaptic sensory neurons. Such qualitative differences are suggestive of motoneuron-type-specific regulation of connectivity.

Connectivity between Identified Neurons Shows Considerable Levels of Variability

We were struck by how variable connectivity between identified neurons seems to be. For example, the number of putative synapses between $IN_{lateral}$ and RP2 motoneurons ranged from 0 to 3 at 0 hr ALH and 6 to16 at 48 hr ALH (Figure 1H). Similarly, for the sensory-motor connection, only a fraction of RP2 and aCC motoneurons receive putative synaptic contacts from dda sensory terminals (Figure 1I). Here, differences in connectivity are mirrored by the diverse routes by which individual neurons attain their connections (Figure 3). For instance, aCC motoneurons form putative synaptic connections with dda sensory axon terminals in every possible way: with contralateral, ipsilateral, or both groups of sensory projections, established by different routes, with dendrites from the main arbor or the soma. This shows that postsynaptic dendritic arbors of motoneurons are quite flexible in how they attain connections with presynaptic terminals.

Local Interactions between Partner Cells Underlie Connectivity Patterns

Next, we inquired into possible causes for the variable connectivity that we see. There is no clear indication that the connectivity we have been able to measure becomes progressively more reproducible as the network matures (Figure 1H). We then asked whether differences in segmental identity contributed to the variability we see. Regression analyses show no statistically significant link between the segmental identity of RP2 and aCC motoneurons and the number of putative synapses that these receive from the $IN_{lateral}$ at 0 hr ALH, 24 hr ALH, or 48 hr ALH (Figure S4).

Next, we considered the effects that local and global network adjustments might have on connectivity. To this end, we focused on pairs of RP2 and aCC neurons located in the same nerve cord and connected to the same $IN_{lateral}$ and

synapses with the $IN_{lateral}$ at 24 hr and 48 hr as compared to aCC contralateral arbors: ANOVA (p values = 0.06 and 0.01) with uncorrected Fisher's least significant difference test, *p = 0.0415 and **p = 0.0083, respectively.

(I) Motoneuron-dda connectivity or absence thereof is shown. RP2 and aCC differ significantly in their sensory-motor connectivity, at 24 hr and 48 hr (Fisher's exact test, **p = 0.0023 and **p = 0.0098, respectively).

Anterior is up in (A)–(E); dorsal is up in (F)–(G′). Dashed line represents the midline. Scale bars represent 10 μm, except in (E), where each inset is 5 × 5 μm. See Figures S1–S3 for tests of GRASP and Brp::mRFP for reporting on synapses.

asked whether having a common presynaptic partner leads to more similar numbers of synapses formed with the same axon. We find that RP2 and aCC motoneurons can vary substantially in the number of putative connections they receive from the same presynaptic partner (Figure 4A). These data imply that local interactions between individual pairs of neurons, rather than global network effects, might determine the outcome of connectivity.

In summary, these observations suggest that variability in connectivity might be an inherent feature of this motor network, at least for the cells analyzed here.

Presynaptic Sites Are Randomly Distributed and Not Predictive of Connectivity

Since synapses are the product of interactions between presynaptic and postsynaptic terminals, we wondered whether the variability we observe arises from one or the other synaptic partner. Testing the potential for an instructive role by the presynaptic interneuron, we asked whether there was any pattern to the distribution of presynaptic sites along the $IN_{lateral}$ axon. We found that along the $IN_{lateral}$ axon (segments A2 to A8), the number of presynaptic sites per neuron was highly variable, ranging from 48 to 107 (85 ± 16.8, SD, n = 17). At the same time, the distribution of presynaptic sites and the spacing between these are indistinguishable from random (Figures 4B and 4C). Thus, we see no evidence of positional patterning of en passant presynaptic sites along $IN_{lateral}$ axons, which has been observed in other systems [25–27].

We then asked whether differences in presynapse number could explain the variability in connectivity between different $IN_{lateral}$-motoneuron pairs. To this end, we correlated for each $IN_{lateral}$-motoneuron pair the number of putative synapses formed with the local density of "available" presynaptic Brp::mRFP puncta located within the $IN_{lateral}$ axon along the span of the motoneuron dendritic tree. We found no significant correlation (Pearson's r = 0.49, p = 0.15, n = 10) (Figure S5). This suggests that, at least in this system, the density of available presynaptic sites is not predictive of how many synaptic connections are formed with the postsynaptic motoneuron. Instead, these data are compatible with a model where the

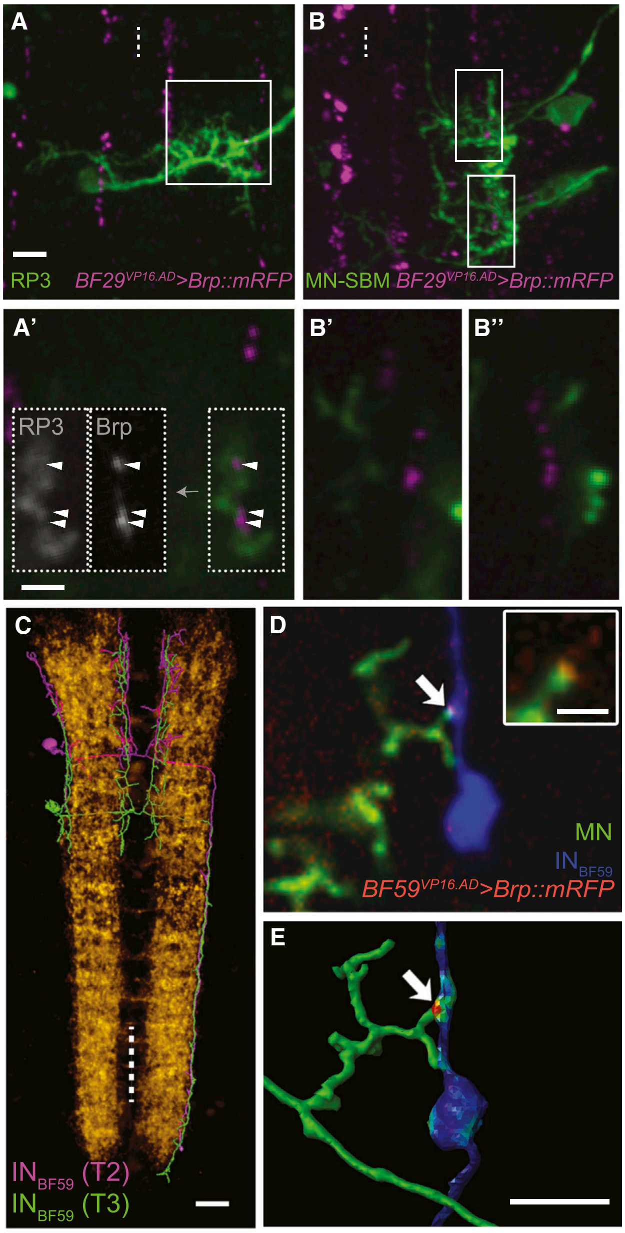

F

Motoneuron type	Frequency at which synaptic contacts with IN_{BF59} were observed
aCC	5 / 6
RP2	3 / 3
LL1	5 / 7
RP3	6 / 12

Figure 2. Variable Connectivity between Intersegmental Interneurons and Motoneurons

(A–B″) Comparison of connectivity between an interneuron from the split-Gal4 expression line *BF29*$^{VP16.AD}$ ($IN_{lateral}$) and two motoneuron types: RP3, innervating longitudinal muscles, and the motor neuron of the segment border muscle (MN-SBM), innervating the transverse segment border muscle (SBM). Z projections of confocal image sub-stacks show presynaptic sites reported by *UAS-brp::mRFP* (magenta) and motoneurons manually labeled with DiD (green). (A′), (B′), and (B″) show the insets in (A) and (B) in more detail, as single confocal planes. The gray level insets in (A′) show the Brp::mRFP and DiD motorneuron channels separately. Although RP3 and the MN-SBM cover similar dendritic areas around the $IN_{lateral}$, RP3 shows putative contacts (arrowheads), whereas the MN-SBM does not.

(C) First instar larval nerve cord with neuropil visualized with alpha-Bungarotoxin-Alexa Fluor 488 (yellow) and two reconstructions of intersegmental interneurons from the split-Gal4 expression line *BF59*$^{VP16.AD}$ intersected with *Cha(7.4kb)*$^{Gal4.DBD\ J8A1}$, shown in thoracic segments T2 (magenta) and T3 (green).

(D and E) Putative synaptic contacts between motoneurons and these intersegmental interneurons (IN_{BF59}) at larval hatching. Interneurons expressing *UAS-brp::mRFP* (red) were manually labeled with DiO (blue), and motoneurons were visualized with DiD (green).

(D) Single confocal optical section.

(E) Reconstructed interneuron (blue) and partially reconstructed dendritic arbor of motoneuron (MN, green) where a site of likely physical overlap coinciding with a presynaptic site is highlighted red (arrow). The inset in (D) is a single confocal section showing the overlap in more detail in the motoneuron (green) and presynaptic marker (red) channels.

(F) Table of frequencies at which synaptic contacts with BF59$^{VP16.AD}$ interneurons were observed for different motoneuron types.

Anterior is up. Ventral midline is indicated by dashed line. Scale bars of (A), (B), and (C) represent10 μm; scale bars of (A′), (B′), (B″), (D), and (E) represent 5 μm; the scale bar of (D) inset represents 1 μm.

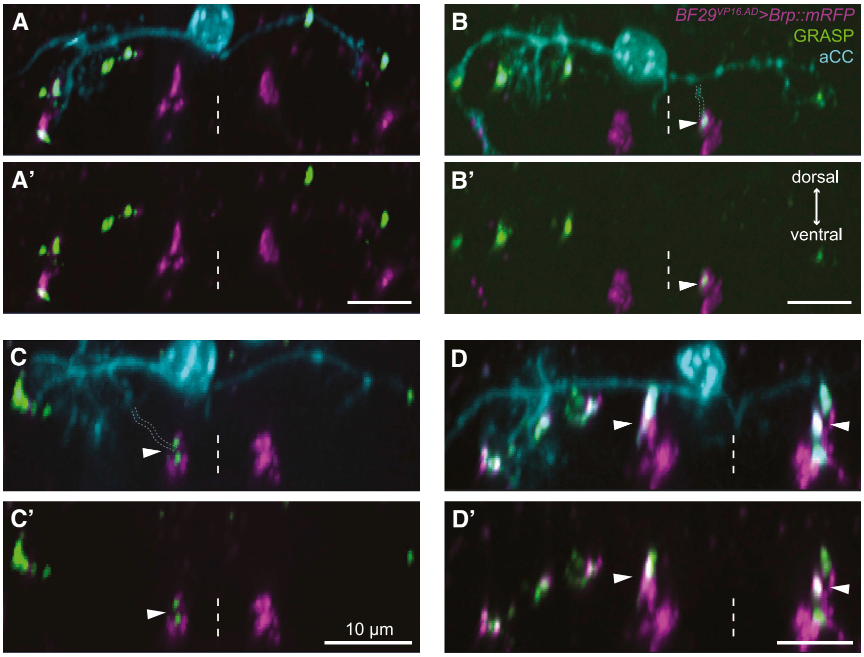

Figure 3. aCC Motor-Sensory Neuron Connections Are Achieved through Different Routes

(A–D′) Cross-section views of nerve cords showing the different configurations of aCC-dda connections (arrowheads): no connection (A), contralateral (B), ipsilateral (C), or bilateral (D). In order to make the distinction among pseudo-colored motoneuron (cyan), GRASP (green), and Brp::mRFP (magenta) easier, the GRASP and Brp channels are also displayed separately in (A′), (B′), (C′), and (D′). In (B) and (C), aCC neurites connecting to the ddas are outlined with dotted lines to show that synaptic dendritic segments do not always originate from the main dendritic tree. Dorsal is up. All scale bars represent 10 μm. Dashed line indicates midline. All data were collected 24 hr ALH.

postsynaptic dendritic arbor regulates the number of connections that it forms [28].

Dendrite Positioning Determines Connectivity

Next, we investigated the role of postsynaptic motoneuron dendrites in determining connectivity. Previously, we showed that postsynaptic dendritic arbors regulate the number of inputs they receive by adjusting dendritic growth [28]. In motor networks, dendritic positioning has been suggested to be important in determining partner choice [29–33]. To investigate the role of dendritic arbor positioning in shaping connectivity, we changed the medio-lateral territories of motoneuron dendrites. Increasing dendritic sensitivity to the midline attractant Netrin, by targeted overexpression of the cognate receptor Frazzled/DCC, shifts RP2 dendrites from principally lateral to more medial neuropil regions. This shift leads to a reduction of laterally positioned dendrites, so that fewer are in proximity to the $IN_{lateral}$ axon, and a concomitant increase of dendrites in the medial neuropil, which is innervated by another interneuron with a medial descending projection (IN_{medial}) (Figure 5). As a result, the proportion of synapses between motoneurons and the $IN_{lateral}$ is drastically reduced, whereas the proportion of synapses with the IN_{medial} is greatly increased, as compared to controls (Figure 5C; t test, $p = 0.0005$ and $p = 0.0194$ for RP2 and aCCi, respectively). Although these observations do not assay for changes in partner choice (RP2 and aCC receive connections from both $IN_{lateral}$ and IN_{medial}), these findings are compatible with a model where connections in motor systems emerge, to an extent, as a consequence of geographical overlap between presynaptic and postsynaptic terminals [30, 32].

In summary, our data point to the existence of mechanisms that allow postsynaptic neurons to determine in a cell-type-specific fashion the number of presynaptic synapses they accept. Clearly, geographical overlap between presynaptic and postsynaptic terminals is necessary for synaptic connections to form, and our experiments suggest that dendritic positioning mechanisms contribute to the emergence of connectivity.

Discussion

There is currently no consensus among views on how patterns of connections develop in a motor network. On the one hand, a great deal of genetically encoded specificity is evident in parts of the mouse spinal cord. For example, group 1a afferents target motoneuron pools with accuracy, and their connectivity is buffered, so that normal information flow is largely maintained in the face of considerable disturbances [34]. Precision of wiring is perhaps most explicit in the selective positioning of inhibitory synapses by the so-called GABA pre-interneurons onto terminals of proprioceptive 1a sensory afferents. This precise and apparently invariant wiring is mediated by the expression of at least two sets of complementary heterophilic transsynaptic cell adhesion molecules [35, 36]. Contrasting with this view are studies from *Xenopus* tadpoles, where two-electrode recordings unequivocally demonstrated a surprising lack of specificity in synaptic connections during early stages of motor network development. Modeling based on these observations further suggests that such rather non-specific wiring patterns are able to generate swimming like motor outputs and that those patterns of connectivity could be formed simply through geographical overlap of coarsely defined presynaptic and postsynaptic termination zones [30, 32]. A limitation in those studies is that they look at groups of similar cells; this has precluded detailed insights at the level of individual synapses over developmental time. Here, we worked with identified partner neurons and studied how synaptic patterns in a motor network change, as the animal develops and grows.

Connectivity Is Consolidated during Development

A striking observation from this study is that at the output face of the network, motoneurons increase synaptic contacts with existing presynaptic partners over time (Figures 1H and 1I). This correlates with previous observations that synaptic drive also increases during this period of larval development, although we do not yet have a physiological readout for the specific anatomical changes we detailed in this study. For motoneurons, the observed strengthening of existing connections is likely an adaptive mechanism that maintains the ability to effectively depolarize muscles as they enlarge during development [17]. Although we have not been able to assay for addition of new presynaptic partners during development,

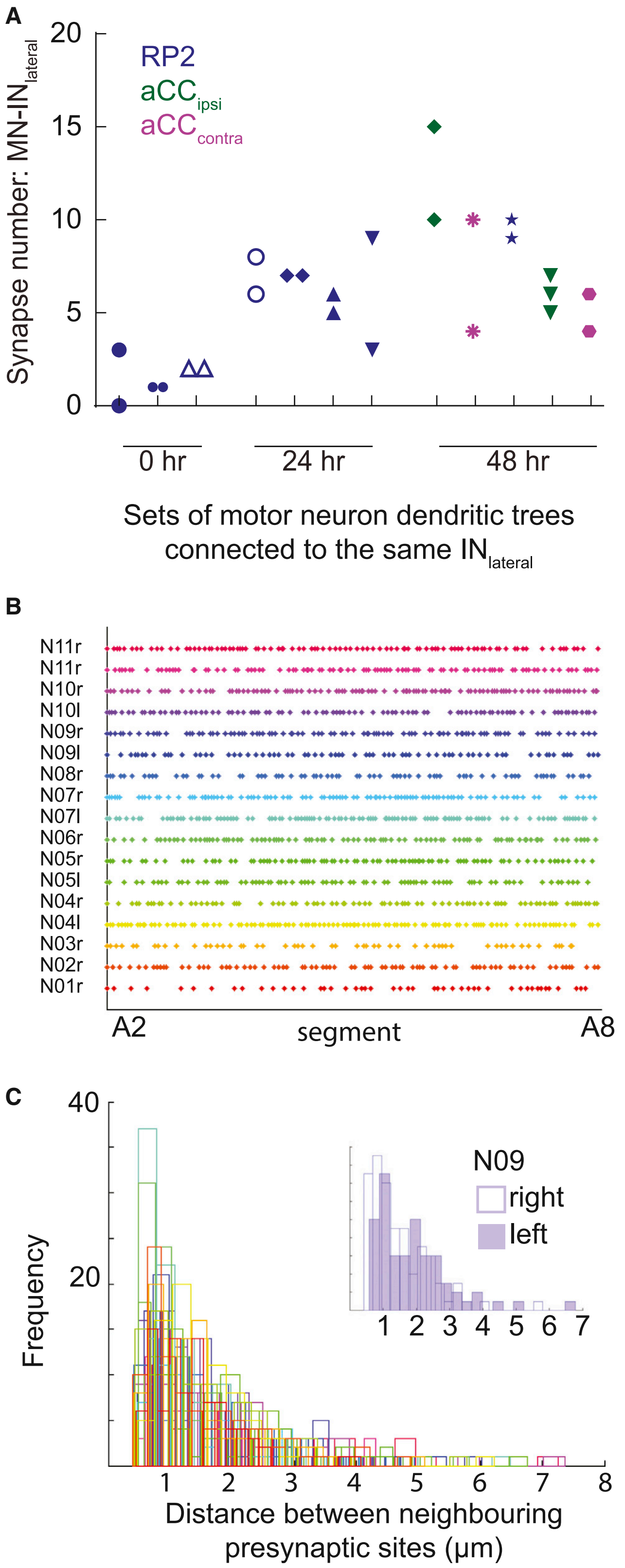

Figure 4. Presynaptic Sites Appear Randomly Distributed along the IN$_{lateral}$ Axon and Do Not Predetermine Connectivity Outcomes

(A) Two or more motoneurons of the same kind (RP2, aCC ipsilateral, or aCC contralateral dendritic arbors) can receive different numbers of synaptic connections from the same presynaptic IN$_{lateral}$ axon. The number of synapses does not correlate with the antero-posterior location of the motoneuron (see Figure S4). Observations from different larval stages are displayed.

(B) Distribution of *UAS-brp::mRFP*-labeled presynaptic sites along the IN$_{lateral}$ axon traversing segments A2 to A8. Data are shown for 17 neurons from 11 individuals with axons normalized for length. Brp::mRFP puncta distribution along axons was indistinguishable from random (Kolmogorov-Smirnov two-sample test, $p = 0.89$).

(C) Distribution of distances between adjacent presynaptic sites shown in (B) is also indistinguishable from random. Inset in (C): distribution of distances between adjacent presynaptic sites in the IN$_{lateral}$ homologs within one specimen (left is indicated by open bars; right is indicated by filled bars). Differences in local Brp::mRFP puncta densities do not correlate with the number of synapses made onto adjacent motoneurons (see Figure S5).

this wiring strategy contrasts with those proposed for cortical neurons, where pyramidal cells are thought to maximize the diversity of presynaptic inputs while keeping synapse number with each partner at a minimum [37].

Connectivity in the Motor System Is Cell Specific yet Variable

Remarkably, reproducible cell-cell interactions during nervous system development can be genetically encoded, and this has been most clearly demonstrated with identified nerve cells of invertebrates—from highly specific substrate choices during axon path finding [38] to the selection of synaptic partners and the number of synapses formed [10, 39]. In the *Drosophila* larval motor system, we find that different motoneuron types have characteristic patterns of connections. For example, the likelihood of forming connections with the proprioceptive dda sensory neurons differs between the RP2 and aCC motoneurons (Figure 1I). Qualitative differences in the specificity of partner choice are also present in that the IN$_{lateral}$ forms connections with motoneurons that innervate longitudinal body wall muscles (e.g., aCC, RP2, and RP3), but not with motoneurons thought to be antagonistic in operation, despite close proximity of their dendrites [24, 40] (Figure 2).

At the same time, this motor system also manifests a considerable degree of variability, both in the likelihood and the number of connections that form between motor and pre-motor interneurons. Although some connection patterns seem to become more reproducible during early phases of network maturation, such as those between the RP2 motoneuron and dda sensory terminals, by and large, our observations suggest that connectivity is inherently flexible and that it is the outcome of local cell-cell interactions, at least between most cells that we have been able to study. For example, two identical motoneurons (in different neuromeres) contacting the same IN$_{lateral}$ axon can form quite different numbers of putative connections with the same presynaptic cell (Figure 4A). It is conceivable that these connections are variable because they are not critical to motor system operation, and it remains to be seen to what extent the observations of this study are representative of connectivity elsewhere in this network.

Where does the information that determines these connectivity outcomes reside? We found no correlation with segmental identity (Figure S4) or evidence for presynaptic patterning information: the number of presynaptic release sites that any one IN$_{lateral}$ makes varies considerably, both between and within animals (left versus right homolog), and their distribution along the axon appears to be random, yet fairly even, with similar numbers of presynaptic sites per neuromere (Figures 4B and 4C). Most compatible with our data is the

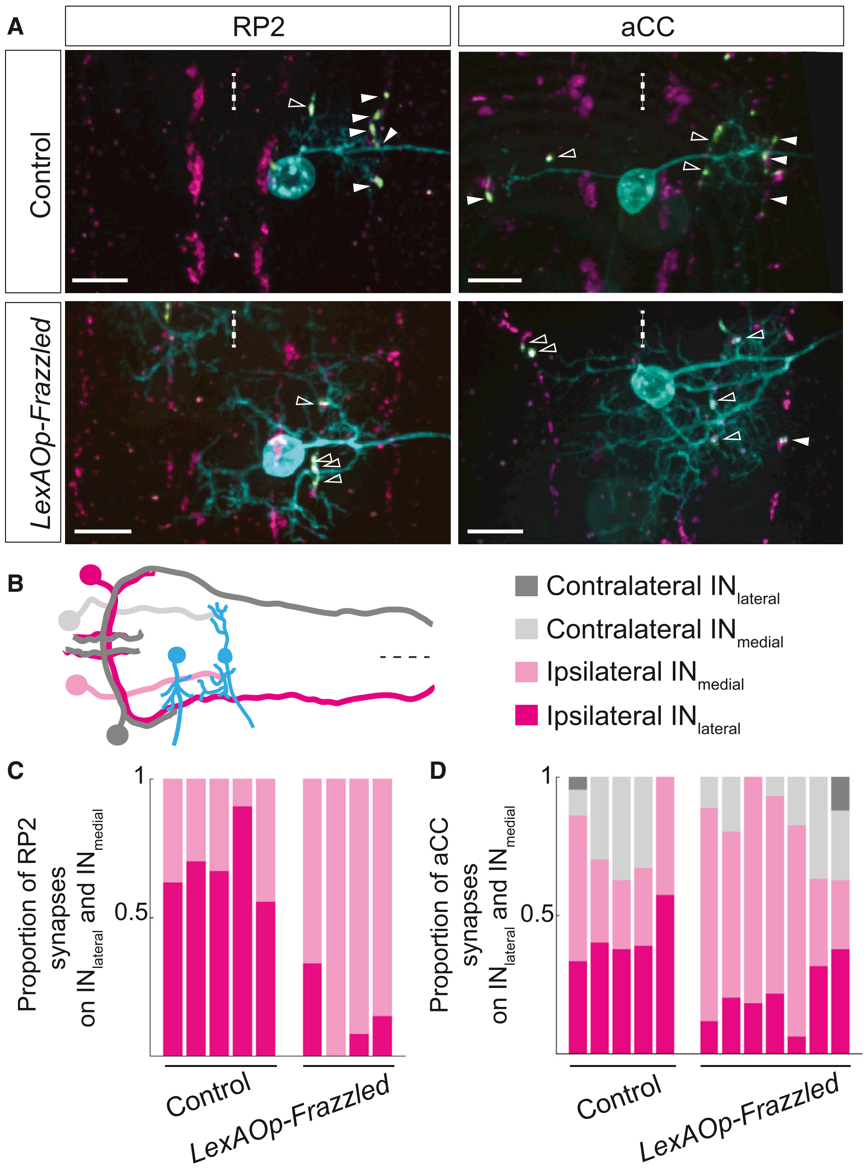

Figure 5. Positioning of Postsynaptic Motoneuron Dendrites Is an Important Factor in Determining the Number of Connections

(A) Changing the medio-lateral positioning of motoneuron dendrites by targeted overexpression of the guidance cue receptor Frazzled/DCC shifts the distribution of the dendritic arbor away from the lateral neuropil (where the IN$_{lateral}$ partner axon is located) and toward the midline, closer to the IN$_{medial}$ axon. Magenta indicates *BF29$^{VP16.AD}$ > brp::mRFP*; cyan indicates motoneurons; green indicates GRASP signal, with arrowheads highlighting contacts with the IN$_{medial}$ (open arrowhead) and IN$_{lateral}$ (filled arrowhead), respectively.

(B) Diagram of motoneuron dendrites (cyan) contacting ipsilateral (magenta) and contralateral (gray) IN$_{lateral}$ axons (darker shade) and IN$_{medial}$ axons (lighter shade).

(C and D) In thoracic segments, the proportion of synaptic sites that motoneurons make with the IN$_{lateral}$ as compared to the IN$_{medial}$ changes with the repositioning of motoneuron dendrites (t test; RP2: $p = 0.0005$; aCCi: $p = 0.0194$).

Scale bars represent 10 μm. Dashed lines in (A) and (B) indicate midline. Anterior is up in (A) and left in (B).

notion that patterns of connectivity are predominantly determined by the postsynaptic dendrites of motoneurons.

Postsynaptic Terminals Regulate Synapse Number

We previously showed that motoneurons achieve a specific range of synaptic input by adjusting the growth of their dendritic arbors [28]. These structural adjustments mirror and complement homeostatic changes of neuronal excitable properties [41, 42]. Here, we show that different dendritic growth patterns lead to different connectivity outcomes. For example, aCC motoneurons are capable of initiating growth of dendritic branches from different parts of the cell, which can form connections with the ipsilateral and/or contralateral dda terminals, or neither (Figure 3). In an analogous situation, in the mouse retina, differences in dendritic growth lead to distinct connection patterns between different bipolar cells and presynaptic photoreceptor terminals [43]. We experimentally tested how dendritic positioning impacts connectivity. Changing the bias so that motoneurons preferentially elaborate their dendrites toward the ventral midline results in changes in connectivity, namely reductions in the proportion of synapses with the lateral IN$_{lateral}$ and concomitant increases in connections with the medially located IN$_{medial}$ axon (Figure 5). Although this experiment does not inform about partner choice, since both the IN$_{lateral}$ and IN$_{medial}$ are normally contacted by these motoneurons, it suggests that the number of connections is determined by the extent to which presynaptic and postsynaptic terminal arbors are targeted to common regions. These experiments in the *Drosophila* larva support observations and models on connectivity in the motor network of *Xenopus* tadpoles, which suggest that the connectivity matrix might be determined in considerable part by geographical overlap of coarsely defined presynaptic and postsynaptic territories [30, 32]. There is evidence that the conserved Slit-Robo and Netrin-Frazzled/DCC guidance cue systems define such territories for positioning axon tracts and regions of dendritic arborization in the CNS and that these can contribute to shaping synaptic connectivity [31, 33, 44–46]. That said, it remains to be established how the promiscuity of connections apparent in early *Xenopus* tadpoles changes over developmental time and to what extent hardwired specificity is genetically encoded elsewhere in the *Drosophila* or indeed in other motor networks.

Experimental Procedures

The complete details of the experimental procedures are provided in the Supplemental Experimental Procedures.

Fly Strains

Motoneurons were visualized using *RN2-Flp^{A},tub84B-FRT-stop-FRT-LexA.VP16, LexAOp-myr::Cerulean* [20]. Cholinergic pre-motor sensory neuron

and interneuron Split-Gal4 expression lines, *BF29*$^{VP16.AD}$ and *BF59*$^{VP16.AD}$, were generated in a Split-Gal4-based enhancer trap screen [18]. Putative synapses were defined as sites where the presynaptic marker Brp::mRFP [21, 31] coincides with physical neuro-neuronal contact indicated by bimolecular fluorescence GRASP [22, 47] (Figures S1 and S3). Here, we use the full-length version of *UAS-brp::mRFP*. Its expression in $IN_{lateral}$ axons is consistent with that of other synaptic markers (Figures S2A–S2C and S2G–S2K) and leads to similar numbers of Brp::XFP puncta as seen when expressing *UAS-brp-short::mStraw* (Figures S2D–S2F) [48].

Dissections, Dye Fills, and Imaging

Nerve cords of staged larvae were dissected in Sørensen's phosphate buffer (0.1 M [pH 7.2]), transferred onto a poly-L-lysine (Sigma) coated coverslip in saline, and imaged immediately with a Yokagawa CSU-22 confocal field scanner mounted on an Olympus BX51WI microscope, using a 63×/1.2 NA (Olympus) water immersion objective. Images were acquired at 0.3-μm z steps using a QuantEM cooled EMCCD camera (Photometrics), operated via MetaMorph software (Molecular Devices). Dye fills were carried out as detailed previously [31].

Supplemental Information

Supplemental Information includes Supplemental Experimental Procedures, five figures, and one movie and can be found with this article online at http://dx.doi.org/10.1016/j.cub.2014.12.056.

Author Contributions

L.C., J.F.E., and M.L. designed the research. L.C., A.S.M., T.Y., S.D., J.F.E., and M.L. performed the research. L.C., A.S.M., J.F.E., and M.L. analyzed the data. L.C., A.S.M., J.F.E., and M.L. wrote the paper.

Acknowledgments

We are grateful to Mathieu Brochet for generating the *13xLexAOp2-frazzled-myc* transgene and mapping the *BF29*$^{VP16.AD}$ insertion; to Ben Fiddian for generating and characterizing *ET*$^{VP16.AD}$ insertions; and to Maarten Zwart and Albert Cardona for generously sharing unpublished observations. We thank Michael Gordon, Kristin Scott, Stephan Sigrist, and Benjamin White for generously providing fly stocks and Hermann Aberle for the anti-DvGlut antiserum. Other fly stocks were obtained from the Bloomington Stock Center. The monoclonal antibodies anti-Fasciclin II 1D4, developed by C. Goodman and colleagues, anti-Sex comb reduced 6H4.1, developed by D. Brower and colleagues, and anti-ChaT 4B1, developed by P.M. Salvaterra and colleagues, were obtained from the Developmental Studies Hybridoma Bank, created by the NICHD of the NIH and maintained at The University of Iowa, Department of Biology. We thank Jimena Berni, members of the M.L. laboratory, Vincent Plagnol, and four reviewers for helpful suggestions. L.C. was supported by a Fyssen Foundation post-doctoral fellowship. This work was supported by a Biotechnology and Biological Sciences Research Council (UK) grant (BB/I022414/1) to M.L., a Wellcome Trust Programme Grant (WT075934) to Michael Bate and M.L., a Grass Foundation fellowship to A.S.M., and a Sir Isaac Newton Trust grant to A.S.M. and M.L. The work benefited from facilities supported by a Wellcome Trust Equipment Grant (WT079204) and contributions by the Sir Isaac Newton Trust in Cambridge.

Received: September 5, 2014
Revised: November 18, 2014
Accepted: December 19, 2014
Published: February 19, 2015

References

1. Kerschensteiner, D., Morgan, J.L., Parker, E.D., Lewis, R.M., and Wong, R.O. (2009). Neurotransmission selectively regulates synapse formation in parallel circuits in vivo. Nature *460*, 1016–1020.
2. Blagburn, J.M., and Bacon, J.P. (2004). Control of central synaptic specificity in insect sensory neurons. Annu. Rev. Neurosci. *27*, 29–51.
3. Takemura, S.Y., Lu, Z., and Meinertzhagen, I.A. (2008). Synaptic circuits of the Drosophila optic lobe: the input terminals to the medulla. J. Comp. Neurol. *509*, 493–513.
4. Murthy, M., Fiete, I., and Laurent, G. (2008). Testing odor response stereotypy in the Drosophila mushroom body. Neuron *59*, 1009–1023.
5. Feller, M.B. (2009). Retinal waves are likely to instruct the formation of eye-specific retinogeniculate projections. Neural Dev. *4*, 24.
6. Chalupa, L.M. (2009). Retinal waves are unlikely to instruct the formation of eye-specific retinogeniculate projections. Neural Dev. *4*, 25.
7. Acebes, A., Martín-Peña, A., Chevalier, V., and Ferrús, A. (2011). Synapse loss in olfactory local interneurons modifies perception. J. Neurosci. *31*, 2734–2745.
8. Briggman, K.L., Helmstaedter, M., and Denk, W. (2011). Wiring specificity in the direction-selectivity circuit of the retina. Nature *471*, 183–188.
9. Canal, I., Fariñas, C.I., Gho, M., and Ferrús, A. (1994). The presynaptic cell determines the number of synapses in the Drosophila optic ganglia. Eur. J. Neurosci. *6*, 1423–1431.
10. Hiesinger, P.R., Zhai, R.G., Zhou, Y., Koh, T.W., Mehta, S.Q., Schulze, K.L., Cao, Y., Verstreken, P., Clandinin, T.R., Fischbach, K.F., et al. (2006). Activity-independent prespecification of synaptic partners in the visual map of Drosophila. Curr. Biol. *16*, 1835–1843.
11. Thum, A.S., Leisibach, B., Gendre, N., Selcho, M., and Stocker, R.F. (2011). Diversity, variability, and suboesophageal connectivity of antennal lobe neurons in D. melanogaster larvae. J. Comp. Neurol. *519*, 3415–3432.
12. Chou, Y.-H., Spletter, M.L., Yaksi, E., Leong, J.C.S., Wilson, R.I., and Luo, L. (2010). Diversity and wiring variability of olfactory local interneurons in the Drosophila antennal lobe. Nat. Neurosci. *13*, 439–449.
13. Masuda-Nakagawa, L.M., Tanaka, N.K., and O'Kane, C.J. (2005). Stereotypic and random patterns of connectivity in the larval mushroom body calyx of Drosophila. Proc. Natl. Acad. Sci. USA *102*, 19027–19032.
14. Caron, S.J., Ruta, V., Abbott, L.F., and Axel, R. (2013). Random convergence of olfactory inputs in the Drosophila mushroom body. Nature *497*, 113–117.
15. Aberle, H., Haghighi, A.P., Fetter, R.D., McCabe, B.D., Magalhães, T.R., and Goodman, C.S. (2002). wishful thinking encodes a BMP type II receptor that regulates synaptic growth in Drosophila. Neuron *33*, 545–558.
16. Davis, G.W. (2013). Homeostatic signaling and the stabilization of neural function. Neuron *80*, 718–728.
17. Zwart, M.F., Randlett, O., Evers, J.F., and Landgraf, M. (2013). Dendritic growth gated by a steroid hormone receptor underlies increases in activity in the developing Drosophila locomotor system. Proc. Natl. Acad. Sci. USA *110*, E3878–E3887.
18. Luan, H., and White, B.H. (2007). Combinatorial methods for refined neuronal gene targeting. Curr. Opin. Neurobiol. *17*, 572–580.
19. Baines, R.A., Uhler, J.P., Thompson, A., Sweeney, S.T., and Bate, M. (2001). Altered electrical properties in Drosophila neurons developing without synaptic transmission. J. Neurosci. *21*, 1523–1531.
20. Singh, A.P., Das, R.N., Rao, G., Aggarwal, A., Diegelmann, S., Evers, J.F., Karandikar, H., Landgraf, M., Rodrigues, V., and Vijayraghavan, K. (2013). Sensory neuron-derived eph regulates glomerular arbors and modulatory function of a central serotonergic neuron. PLoS Genet. *9*, e1003452.
21. Wagh, D.A., Rasse, T.M., Asan, E., Hofbauer, A., Schwenkert, I., Dürrbeck, H., Buchner, S., Dabauvalle, M.C., Schmidt, M., Qin, G., et al. (2006). Bruchpilot, a protein with homology to ELKS/CAST, is required for structural integrity and function of synaptic active zones in Drosophila. Neuron *49*, 833–844.
22. Feinberg, E.H., Vanhoven, M.K., Bendesky, A., Wang, G., Fetter, R.D., Shen, K., and Bargmann, C.I. (2008). GFP Reconstitution Across Synaptic Partners (GRASP) defines cell contacts and synapses in living nervous systems. Neuron *57*, 353–363.
23. Cheng, L.E., Song, W., Looger, L.L., Jan, L.Y., and Jan, Y.N. (2010). The role of the TRP channel NompC in Drosophila larval and adult locomotion. Neuron *67*, 373–380.
24. Heckscher, E.S., Lockery, S.R., and Doe, C.Q. (2012). Characterization of Drosophila larval crawling at the level of organism, segment, and somatic body wall musculature. J. Neurosci. *32*, 12460–12471.
25. Shen, K., Fetter, R.D., and Bargmann, C.I. (2004). Synaptic specificity is generated by the synaptic guidepost protein SYG-2 and its receptor, SYG-1. Cell *116*, 869–881.
26. Ango, F., Wu, C., Van der Want, J.J., Wu, P., Schachner, M., and Huang, Z.J. (2008). Bergmann glia and the recognition molecule CHL1 organize GABAergic axons and direct innervation of Purkinje cell dendrites. PLoS Biol. *6*, e103.
27. Li, L., Tasic, B., Micheva, K.D., Ivanov, V.M., Spletter, M.L., Smith, S.J., and Luo, L. (2010). Visualizing the distribution of synapses from individual neurons in the mouse brain. PLoS ONE *5*, e11503.

28. Tripodi, M., Evers, J.F., Mauss, A., Bate, M., and Landgraf, M. (2008). Structural homeostasis: compensatory adjustments of dendritic arbor geometry in response to variations of synaptic input. PLoS Biol. *6*, e260.
29. Brierley, D.J., Blanc, E., Reddy, O.V., Vijayraghavan, K., and Williams, D.W. (2009). Dendritic targeting in the leg neuropil of *Drosophila*: the role of midline signalling molecules in generating a myotopic map. PLoS Biol. *7*, e1000199.
30. Li, W.-C., Cooke, T., Sautois, B., Soffe, S.R., Borisyuk, R., and Roberts, A. (2007). Axon and dendrite geography predict the specificity of synaptic connections in a functioning spinal cord network. Neural Dev. *2*, 17.
31. Mauss, A., Tripodi, M., Evers, J.F., and Landgraf, M. (2009). Midline signalling systems direct the formation of a neural map by dendritic targeting in the Drosophila motor system. PLoS Biol. *7*, e1000200.
32. Roberts, A., Conte, D., Hull, M., Merrison-Hort, R., al Azad, A.K., Buhl, E., Borisyuk, R., and Soffe, S.R. (2014). Can simple rules control development of a pioneer vertebrate neuronal network generating behavior? J. Neurosci. *34*, 608–621.
33. Godenschwege, T.A., Simpson, J.H., Shan, X., Bashaw, G.J., Goodman, C.S., and Murphey, R.K. (2002). Ectopic expression in the giant fiber system of Drosophila reveals distinct roles for roundabout (Robo), Robo2, and Robo3 in dendritic guidance and synaptic connectivity. J. Neurosci. *22*, 3117–3129.
34. Pecho-Vrieseling, E., Sigrist, M., Yoshida, Y., Jessell, T.M., and Arber, S. (2009). Specificity of sensory-motor connections encoded by Sema3e-Plxnd1 recognition. Nature *459*, 842–846.
35. Betley, J.N., Wright, C.V., Kawaguchi, Y., Erdélyi, F., Szabó, G., Jessell, T.M., and Kaltschmidt, J.A. (2009). Stringent specificity in the construction of a GABAergic presynaptic inhibitory circuit. Cell *139*, 161–174.
36. Ashrafi, S., Betley, J.N., Comer, J.D., Brenner-Morton, S., Bar, V., Shimoda, Y., Watanabe, K., Peles, E., Jessell, T.M., and Kaltschmidt, J.A. (2014). Neuronal Ig/Caspr recognition promotes the formation of axoaxonic synapses in mouse spinal cord. Neuron *81*, 120–129.
37. Wen, Q., Stepanyants, A., Elston, G.N., Grosberg, A.Y., and Chklovskii, D.B. (2009). Maximization of the connectivity repertoire as a statistical principle governing the shapes of dendritic arbors. Proc. Natl. Acad. Sci. USA *106*, 12536–12541.
38. Raper, J.A., Bastiani, M.J., and Goodman, C.S. (1984). Pathfinding by neuronal growth cones in grasshopper embryos. IV. The effects of ablating the A and P axons upon the behavior of the G growth cone. J. Neurosci. *4*, 2329–2345.
39. Murphey, R.K., and Lemere, C.A. (1984). Competition controls the growth of an identified axonal arborization. Science *224*, 1352–1355.
40. Landgraf, M., Jeffrey, V., Fujioka, M., Jaynes, J.B., and Bate, M. (2003). Embryonic origins of a motor system: motor dendrites form a myotopic map in Drosophila. PLoS Biol. *1*, E41.
41. Baines, R.A., and Bate, M. (1998). Electrophysiological development of central neurons in the Drosophila embryo. J. Neurosci. *18*, 4673–4683.
42. Marder, E., Tobin, A.E., and Grashow, R. (2007). How tightly tuned are network parameters? Insight from computational and experimental studies in small rhythmic motor networks. Prog. Brain Res. *165*, 193–200.
43. Dunn, F.A., and Wong, R.O.L. (2012). Diverse strategies engaged in establishing stereotypic wiring patterns among neurons sharing a common input at the visual system's first synapse. J. Neurosci. *32*, 10306–10317.
44. Simpson, J.H., Bland, K.S., Fetter, R.D., and Goodman, C.S. (2000). Short-range and long-range guidance by Slit and its Robo receptors: a combinatorial code of Robo receptors controls lateral position. Cell *103*, 1019–1032.
45. Sabatier, C., Plump, A.S., Le Ma, Brose, K., Tamada, A., Murakami, F., Lee, E.Y., and Tessier-Lavigne, M. (2004). The divergent Robo family protein rig-1/Robo3 is a negative regulator of slit responsiveness required for midline crossing by commissural axons. Cell *117*, 157–169.
46. Rajagopalan, S., Vivancos, V., Nicolas, E., and Dickson, B.J. (2000). Selecting a longitudinal pathway: Robo receptors specify the lateral position of axons in the Drosophila CNS. Cell *103*, 1033–1045.
47. Gordon, M.D., and Scott, K. (2009). Motor control in a Drosophila taste circuit. Neuron *61*, 373–384.
48. Christiansen, F., Zube, C., Andlauer, T.F., Wichmann, C., Fouquet, W., Owald, D., Mertel, S., Leiss, F., Tavosanis, G., Luna, A.J., et al. (2011). Presynapses in Kenyon cell dendrites in the mushroom body calyx of Drosophila. J. Neurosci. *31*, 9696–9707.

Current Biology
Supplemental Information

Development of Connectivity in a Motoneuronal Network in *Drosophila* Larvae

Louise Couton, Alex S. Mauss, Temur Yunusov, Soeren Diegelmann, Jan Felix Evers, and Matthias Landgraf

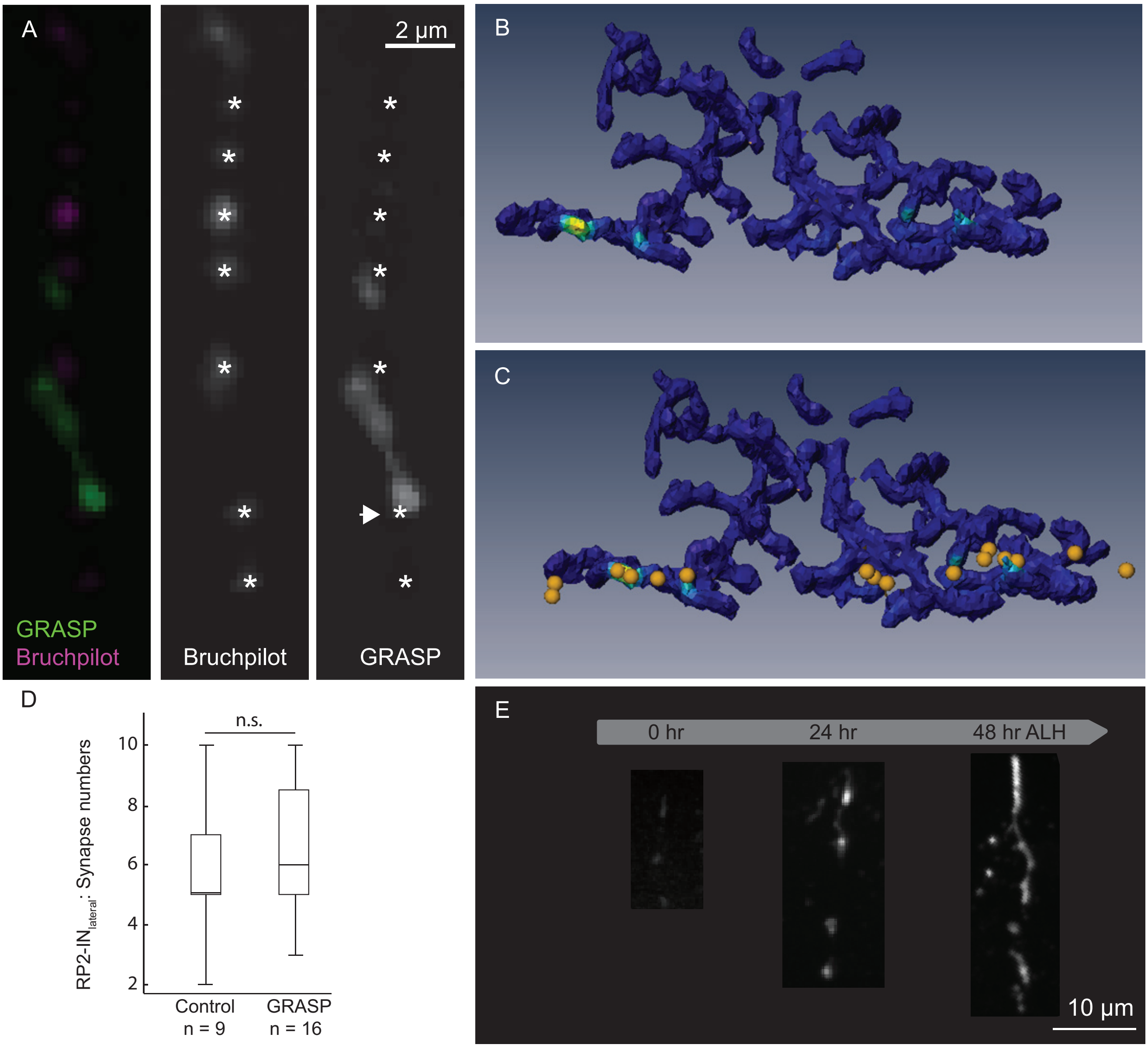
A
GRASP
Bruchpilot
Bruchpilot
2 µm
GRASP
B
C
D
n.s.
10
8
6
4
2
RP2-IN$_{lateral}$: Synapse numbers
Control
n = 9
GRASP
n = 16
E
0 hr
24 hr
48 hr ALH
10 µm

Figure S1

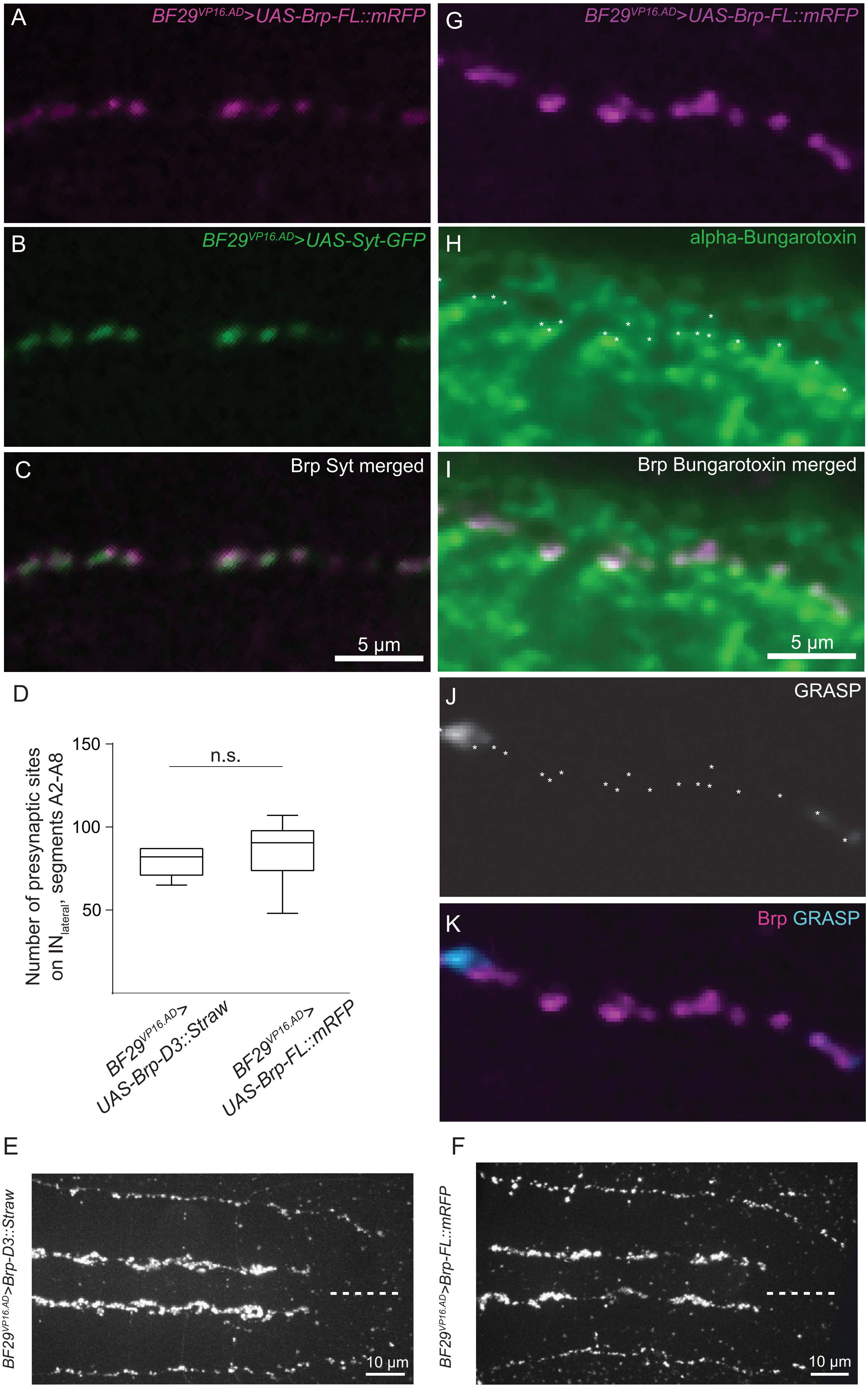
A
BF29VP16.AD>UAS-Brp-FL::mRFP
B
BF29VP16.AD>UAS-Syt-GFP
C
Brp Syt merged
5 µm
D
n.s.
150
100
50
Number of presynaptic sites on INlateral, segments A2-A8
BF29VP16.AD> UAS-Brp-D3::Straw
BF29VP16.AD> UAS-Brp-FL::mRFP
G
BF29VP16.AD>UAS-Brp-FL::mRFP
H
alpha-Bungarotoxin
I
Brp Bungarotoxin merged
5 µm
J
GRASP
K
Brp GRASP
E
BF29VP16.AD>Brp-D3::Straw
10 µm
F
BF29VP16.AD>Brp-FL::mRFP
10 µm

Figure S2

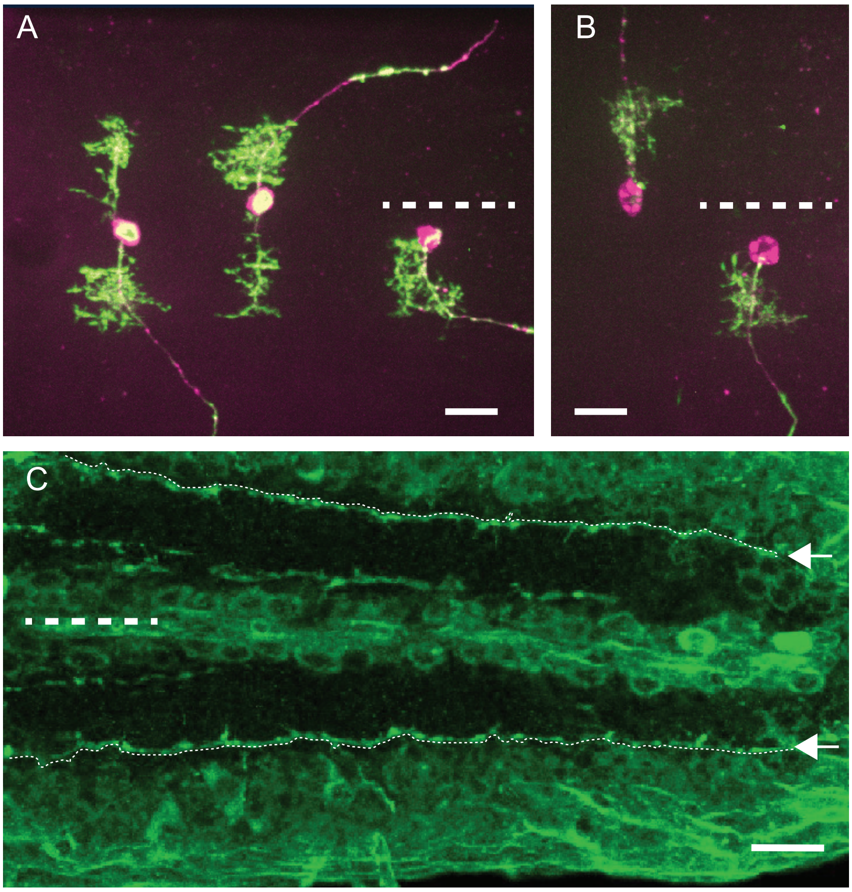

Figure S3

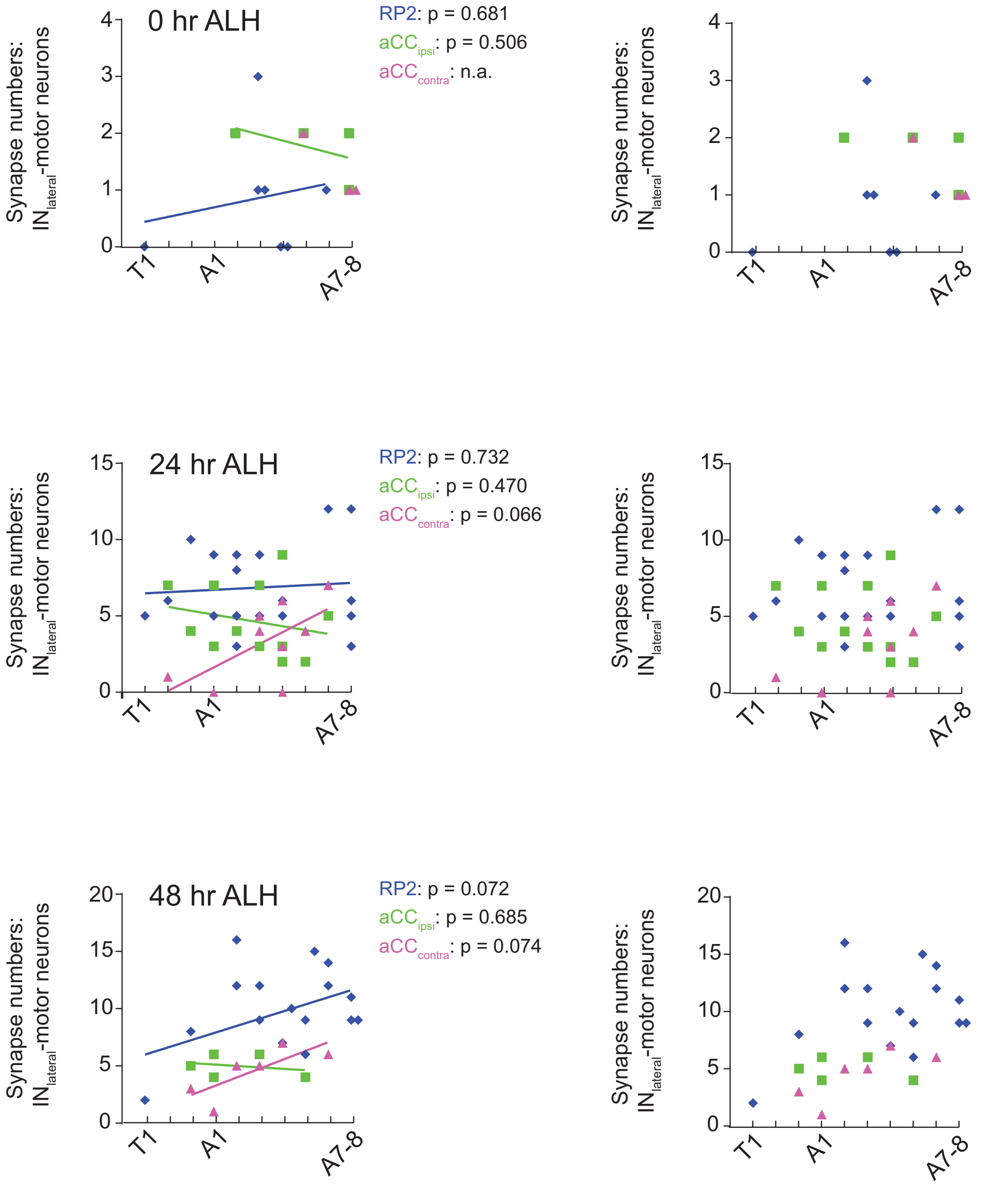
0 hr ALH
RP2: p = 0.681
aCC$_{ipsi}$: p = 0.506
aCC$_{contra}$: n.a.
24 hr ALH
RP2: p = 0.732
aCC$_{ipsi}$: p = 0.470
aCC$_{contra}$: p = 0.066
48 hr ALH
RP2: p = 0.072
aCC$_{ipsi}$: p = 0.685
aCC$_{contra}$: p = 0.074
Synapse numbers: IN$_{lateral}$-motor neurons
T1
A1
A7-8

Figure S4

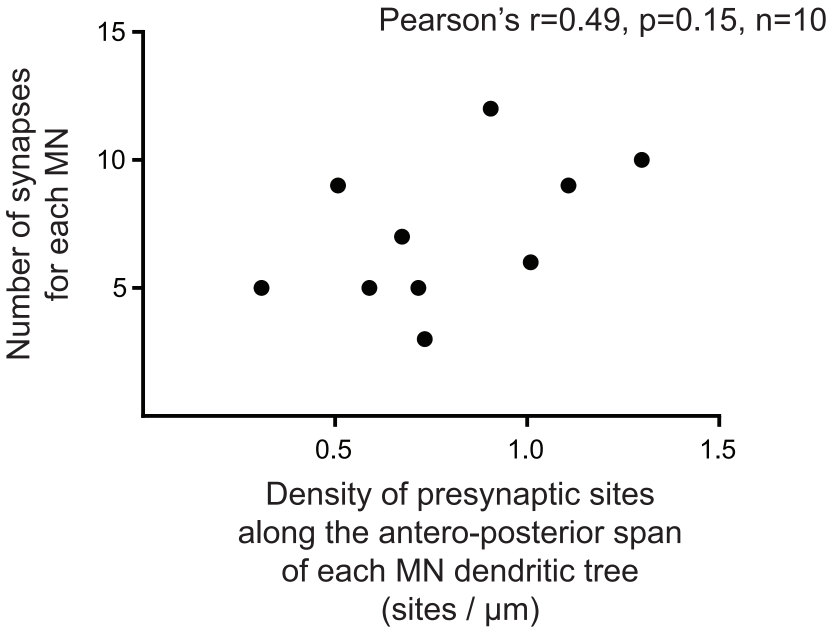
Pearson's r=0.49, p=0.15, n=10
15
10
5
Number of synapses for each MN
0.5
1.0
1.5
Density of presynaptic sites along the antero-posterior span of each MN dendritic tree (sites / µm)

Figure S5

Figure S1 (associated with Figure 1): GFP reconstitution does not manifestly bias synaptogenesis.

(A) Representative example showing GRASP is not systematically associated with synapses. In spite of an extensive GRASP signal (green), overlap with presynaptic Brp::mRFP puncta (magenta, asterisks) is only found at one site (arrowhead). (B and C) Partially reconstructed motoneuron dendritic tree with (B) and without (C) putative presynaptic sites shown (spheres). Regions where dendrites are closely apposed to presynaptic sites (<300 nm) are highlighted in cyan/yellow. Often dendritic regions are closely apposed to more than one presynaptic site. (D) Comparable numbers of putative synaptic contacts form between RP2 motoneuron dendrites and $IN_{lateral}$ axons with and without GRASP (t-test, p>0.05). Whiskers indicate the highest and lowest value of each dataset. In control experiments one of the GRASP components was omitted and RP2-$IN_{lateral}$ appositions were visualised with the genotype *w-;LexAOp-CD4::spGFP $_{11}$ /+;BF29$^{VP16.AD}$, Cha(7.4kb)$^{Gal4.DBD\ J8A1}$, UAS-brp::mRFP/RN2-Flp^{A}, tub84B-FRT-stop-FRT-LexA.VP16, 13xLexAOp2-IVS- myr::GFP*. RP2 dendritic trees were digitally reconstructed and appositions to $IN_{lateral}$ presynaptic sites within a 300nm radius were determined. (E) Motoneuron dendrites tend to progressively fasciculate with $IN_{lateral}$ axons as larvae grow older, producing a stripe-like GRASP signal along the $IN_{lateral}$ axon. Developmental increases in synapse numbers do not parallel the extent of this increase in the motoneuron dendrite-INlateral axon contact area. Anterior is up in A and E, left in B and C

Figure S2 (associated with Figure 1): Brp::mRFP localises consistently to other pre- and postsynaptic proteins and reports statistically similar numbers of presynaptic sites in the $IN_{lateral}$, compared to *UAS-brp-shortD3-Straw*

(A-C) The products of *UAS-Brp::mRFP* (A) and *UAS-Synaptotagmin::GFP* expressed within the same $IN_{lateral}$ neuron (B) co-localise consistently along the axon (C), suggesting Brp::mRFP is a faithful reporter of presynaptic sites. (D) Box plots depicting the numbers of presynaptic sites reported by each Brp construct in segments A2 to A8 of the $IN_{lateral}$ axon: (*brp-short$^{D3-Straw}$*; n = 6 neurons; *full length-brp::mRFP*; n = 18 neurons; n.s.: not significant, t-test: p>0.05). Whiskers indicate the highest and lowest value of each dataset. (E,F) Dorsal projections of confocal image stacks showing the patterns of expression of *BF29$^{VP16.AD}$*, *Cha(7.4kb)$^{Gal4.DBD\ J8A1}$* driving *UAS-brp-short$^{D3-Straw}$* (E) and *UAS-brp::mRFP* (full length) (F). (G-K) Brp::mRFP puncta along the $IN_{lateral}$ (G) and α-bungarotoxin-Alexa Fluor 647 puncta (H) are consistently apposed (I), supporting the idea that the presynaptic Brp::mRFP puncta are matched with postsynaptic acetylcholine receptor clusters. This is also true of GRASP-positive Brp puncta (J,K). Asterisks mark the locations of Brp::mRFP puncta in the α-bungarotoxin and GRASP channels.

Figure S3 (associated with Figure 1): Both split-GFP fragments distribute evenly in pre- and postsynaptic terminals.

(A, B) Within motoneuron dendrites, CD4::sp-GFP_{11} distributes evenly, as shown by the fact that in a background of cholinergic neurons expressing the complementary fragment CD4::sp-$GFP_{1\text{-}10}$ *(Cha7.4-Gal4; UAS-$GFP_{1\text{-}10}$*) [S1], the whole dendritic tree displays reconstituted GFP fluorescence (green) indicative of cell-cell contact with processes of cholinergic neurons, which provide the majority of synaptic input to motoneurons and constitute the vast majority of cells in the nerve cord. Magenta: RP2 and aCC motoneurons as visualised with *w- ; LexAOp-CD4::spGFP$_{11}$; RN2-Flp^{A}, tub84B-FRT-stop- FRT-LexA.VP16, LexAOp-myr::mCherry*. Confocal image stacks are shown as dorsal maximal z- projections, (but see also the movie corresponding to stack shown as panel B: **Movie S1**). (C) Within the $IN_{lateral}$, CD4::sp-$GFP_{1\text{-}10}$ distributes evenly along the axon (arrows) as visualised with anti-GFP staining (Chicken anti-GFP, Abcam, dilution 1:10000), medially to the thin dotted outline on this panel. Unspecific background staining is seen in this instance on the surface of other cells along the midline and the lateral cortex. Genotype: *w-;+;BF29$^{VP16.AD}$, Cha(7.4kb)$^{Gal4.DBD\ J8A1}$, UAS-Brp::mRFP/UAS- GFP$_{1\text{-}10}$*. Scalebars: 10 µm, dashed lines: midline. Anterior is left.

Figure S4 (associated with Figure 4): The number of motoneuron-$IN_{lateral}$ synapses does not correlate with motoneuron segmental identity.

(A-C) Number of motoneuron-$IN_{lateral}$ synapses plotted against motoneuron segmental location, at 0 hr, 24 hr and 48 hr after larval hatching. Data are shown with (A-C) or without (A'-C') their linear regression lines. None of the regression lines differ significantly from a null slope, indicating that along the antero-posterior axis there are no significant segmental differences in the number of putative synapses formed between the RP2 and aCC motoneurons and the $IN_{lateral}$ interneuron.

Figure S5 (associated with Figure 4): The number of synapses within individual pairs of RP2-$IN_{lateral}$ does not correlate significantly with the local density of presynaptic sites.

The local density of presynaptic sites was calculated as the number of presynaptic sites/ µm along the stretch of $IN_{lateral}$ axon between the anterior-most and posterior-most branches of an RP2 dendritic tree. For each interneuron-motoneuron pair, this was plotted against the actual number of putative synapses, as reported by co-localisation of Brp::mRFP and cell-cell contacts -the latter indicated by reconstituted-GFP-. Data shown were collected 24 hr ALH.

Movie S1 (associated with Figure 1): Even distribution of CD4::sp-GFP_{11} within motoneuron dendrites.

In a background of cholinergic neurons expressing the complementary fragment CD4::sp-$GFP_{1\text{-}10}$ (*Cha7.4-Gal4; UAS-*$GFP_{1\text{-}10}$) [S1], the whole dendritic tree of these CD4::sp-GFP_{11}-expressing RP2 neurons displays reconstituted GFP fluorescence (green). Other examples are shown in **Figure S3**. Magenta: RP2 motoneurons as visualised with *w- ; LexAOp-CD4::sp-*GFP_{11}*; RN2-FlpA, tub84B-FRT-stop-FRT-LexA.VP16, LexAOp-myr::mCherry*. Arrowheads point to the medio-lateral location of the $IN_{lateral}$ (not visualised here).

Supplemental experimental procedures

Staging larvae:

Flies were reared at 25°C on apple juice agar plates supplemented with yeast paste. Eggs (0-8 hr after egg laying) were incubated 16 hr at 29°C for optimal FLP recombinase activity. Stocks and recombinant chromosomes were generated using standard procedures. Transgenic lines were generated by BestGene Inc (California). Data were collected at three time points: 0 hr (first instar), 24 hr (second instar) and 48 hr ALH (third instar). For the first time point, newly hatched larvae were collected within 1 hour of hatching and used immediately. For the second and third time points, larvae were collected within 2 hours of hatching and transferred to fresh food until dissection, 24 hr or 48 hr later.

Visualisation of motoneurons and putative synaptic connections genotype:

w- ; LexAOp-CD4::sp-GFP_{11}*/ + ;* $BF29^{VP16.AD}$*,* $Cha(7.4kb)^{Gal4.DBD\ J8A1}$*, UAS-brp::mRFP /* $RN2\text{-}Flp^{A}$*, tub84B-FRT-stop-FRT-LexA.VP16, LexAOp-myr::Cerulean, UAS-CD4::*$spGFP_{1\text{-}10}$

Split-Gal4 expression pattern and distribution of presynaptic sites analysis genotype:

A balanced line carrying the $BF29^{VP16.AD}$ or $BF59^{VP16.AD}$ insertion was crossed to either *w- ; + ;* $Cha(7.4kb)^{Gal4.DBD\ J8A1}$*, UAS-EGFP* or *w- ; UAS-pmVenus/CyO ;* $Cha(7.4kb)^{Gal4.DBD\ J8A1}$*, UAS-brp::mRFP (full length)*

Dye fills

Freshly hatched larvae were dissected with ventral nerve cord and muscle field kept intact, as described by Baines and Bate [S2], then fixed with 3.7% formaldehyde for 10 min. Interneurons

identified by BF59-driven GFP expression, were impaled with sharp microelectrodes with 2mg/ml Neuro-DiO (Biotium) in EthOH and filled by delivering a hyperpolarizing current via an iontophoretic Dye Marker (Digitimer). Motor neurons were retrogradely labelled with DiD (Life Technologies) or Neuro-DiO (Biotium), dissolved in vegetable oil at 2mg/ml, as described by Landgraf, Bossing et al. [S3].

Image analysis

Image stacks were analysed using Amira software (FEI Visualization Sciences Group, France). To map the distribution of presynaptic sites along interneuron axons we digitally demarcated segments based on the pattern of sensory axon projections and marked Brp::mRFP puncta in segments A2 to A8, using the Amira landmark tool. We then exported the landmark coordinates to Matlab (The MathWorks, Natick, USA), normalised and analysed these using custom made functions. To assess the number of putative synapses between motor neurons and their presynaptic partners, three channel image stacks showing myr::Cerulean (postsynaptic motor neuron), Brp::mRFP (presynaptic sites) and GRASP GFP cell-cell contact were loaded into Amira software. We marked the locations of all Brp::mRFP puncta and recorded those co-localising with GRASP signal as putative synapses. To assess synapse numbers, we only considered motor neurons located in segments A2 to A6, which are more similar in function as compared to homologues located elsewhere in the nerve cord. However, when addressing the effect of segmental identity (Figure S4), we used a wider range of motoneurons from T1 to A8. Reconstructions of dendritic trees and calculation of proximity to the presynaptic partners were performed using a plugin for Amira developed by J-F Evers [S4, 5].

Statistics

Data were plotted and analyzed using Matlab (The MathWorks, Natick, USA) or Prism software (GraphPad Software). At the $IN_{lateral}$-motoneuron interface (**Figure 1H**), changes in synapse numbers through time were tested for using ANOVA and a post-test for trend. At each time point, differences in synapse number between different types of dendritic arbors were tested for using ANOVA and the uncorrected Fisher's LSD test. At the sensory-motor interface (**Figure 1I**), changes in the proportion of motoneurons making synaptic contact were tested for using a Chi-square test for trend. At each time-point, the proportions of each motoneuron type making contact with the sensory partner were compared using Fisher's exact test. In order to assess whether the presynaptic site distribution differed from random (**Figure 4B and C**), we used Matlab to create a set of 17 virtual axons with the same number of presynaptic sites as our actual sample, though

randomly distributed. We then compared both distributions using the Kolmogorov-Smirnov two sample test. In order to ask whether the distribution of synaptic contacts between $IN_{lateral}$ and IN_{medial} changes when the dendritic trees are shifted towards the midline (relates to **Figure 5C and D**), we compared the proportion of synapses located on the $IN_{lateral}$ in the control and frazzled-expressing groups: t-tests were applied to RP2, $aCC_{ipsilateral}$ and $aCC_{contralateral}$ For each of these dendritic trees, we only considered the set of interneuron axons located in the same hemisegment, since the change of dendritic territory is not such that these trees could make connections in the contralateral hemisegment. We used t-tests to compare the average numbers of synapses reported with and without GRASP (**Figure S1**), as well as to compare the numbers of presynaptic sites reported by *UAS-Brp-Straw-D3* and *UAS-Brp::mRFP* (**Figure S2**). We applied a regression analysis to check whether the changes in synapse numbers follow an antero-posterior trend (**Figure S4**). Finally, we used Pearson's R to ask about a possible correlation between the local density of presynaptic sites and the actual number of synapses (**Figure S5**).

Immunocytochemistry

The location of interneurons in *$BF29_{VP16.AD}$* was determined by staining nerve cords against Fasciclin II (mouse anti-Fasciclin II 1D4, 1:20, DSHB) [S6] and Sex comb reduced (mouse anti-Scr 6H4.1, 1:100, DSHB) [S7]. Cholinergic neurotransmitter identity of interneurons in the *BF29VP16.AD* and a *BF59VP16.AD* expression lines were confirmed by staining for mouse anti-ChaT 4B1 (1:100, DSHB) [S8]. Absence of staining for other major transmitters was confirmed by staining with anti-GABA (rabbit anti-GABA 1:1,000 dilution, Sigma) [S9] and anti-DvGlut (rabbit anti-DvGlut 1:500) [S10]. Secondary antibodies were donkey anti-rabbit Cy3 (1:400, Jackson ImmunoResearch), donkey anti-mouse Cy5 (1:400, Jackson ImmunoResearch), donkey anti-mouse CF568 (1:600, Biotium). To label cholinergic receptors, fixed preparations with dye-labeled neurons were incubated with alpha-Bungarotoxin-A488 (2µM) in saline for a few hours. Alternatively, when testing whether Brp::mRFP puncta localised consistently with (postsynaptic) cholinergic receptors, a 2µM solution of alpha-Bungarotoxin-A647 (Life Technologies) was applied for 10 minutes on freshly dissected, unfixed nerve cords expressing Brp::mRFP, rinsed twice for 2 minutes in physiological saline and imaged immediately.

Molecular biology

Mapping the $BF29^{VP16.AD}$ insertion: The genomic insertion site of *$ET^{VP16.AD}$* in the *$BF29^{VP16.AD}$* enhancer-trap line was determined by inverse PCR and mapped to the *eyegone* locus, using the protocol published on the BDGP website. Briefly, genomic DNA isolated from a balanced stock

that only carried the $BF29^{VP16.AD}$ insertion was digested with Sau3A and MspI (New England Laboratories). Fragments were then self-ligated and amplified by inverse PCR using primers Pry4 and Plw3-1.

Construction of LexAOp-myr::Cerulean: The coding sequence for the fluorophore Cerulean and the myristoylation sequence were PCR amplified using primers suitable for multisite Gateway cloning (Life Technologies) to create pEntry vectors (P1-myr-P5r, P5-Cerulean-P2). These vectors were combined in a two fragment recombination reaction into the pLOT-W vector [S11] to fuse the myr::Cerulean downstream of the LexA operon. DNA was purified using a Qiagen Midi Kit and transgenic lines were generated by BestGene Inc. (Chino Hills, CA, USA).

Construction of 13xLexAOp2-frazzled-myc: cDNA coding for *fraB-6xMyc* [S12] was PCR amplified and via Infusion (Clontech) cloned into a XhoI –XbaI linearised pJFRC19 vector backbone [S13] to generate *p13xLexAOp2-IVS-frazzled-myc*, subsequently integrated via PhiC31-mediated recombination into the Su(Hw)attP6 landing site (2L, cytogenetic map: 24D) (BestGene, California) [S14].

Control for the validity of the GRASP approach

A potential concern with using the GRASP reporter is that the GFP reconstitution might lead to strong cell-cell adhesion, which in turn may lead to artefactual synapse formation between apposed cells. To test this, we carried out control experiments, in which one of the GRASP components was omitted and then imaged the RP2-$IN_{lateral}$ contacts, using the following genotype:

w-;LexAOp-CD4:spGF$_{11}$ */+ ;BF29*$^{VP16.AD}$*, Cha(7.4kb)*$^{Gal4.DBD\ J8A1}$*, UAS-brp::mRFP/RN2-Flp*^{A}*, tub84B-FRT- stop-FRT-LexA.VP16, 13xLexAOp2-IVS-myr::GFP*

We then digitally reconstructed RP2 dendritic trees and identified the $IN_{lateral}$ presynaptic sites closely apposed to RP2 dendrites, within a 300 nm radius. Statistically comparable numbers of presynaptic sites form between RP2 motor and $IN_{lateral}$ interneurons with and without presence of bimolecular fluorescence complementation (t-test, $p>0.05$), suggesting that, at least in this experimental system, GRASP mediated cell-cell adhesion does not obviously lead to the formation and/or maintenance of supernumerary synapses (**Figure S1**). A second argument supporting the validity of the GRASP approach in this study is that motoneuron dendrites tend to

increasingly fasciculate with $IN_{lateral}$ axons as larvae grow older. As a result, the GRASP signal changes from an initially punctate appearance at the first larval instar stage to a linear pattern by the third larval instar. This dramatic increase in cell-cell apposition between partner neurons is not paralleled by steep increases in synapse number; *i.e.* large areas of GRASP signal exist that show no overlap with presynaptic sites. These observations suggest that while the bimolecular fluorescence complementation of the GRASP method may mediate artefactual cell-cell adhesion, this appears to be synaptogenically inert.

Supplemental references

S1. Salvaterra, P.M., and Kitamoto, T. (2001). *Drosophila* cholinergic neurons and processes visualized with Gal4/UAS-GFP. Brain Res Gene Expr Patterns *1*, 73-82.

S2. Baines, R.A., and Bate, M. (1998). Electrophysiological development of central neurons in the *Drosophila* embryo. J Neurosci *18*, 4673-4683.

S3. Landgraf, M., Bossing, T., Technau, G.M., and Bate, M. (1997). The origin, location, and projections of the embryonic abdominal motorneurons of *Drosophila*. J Neurosci *17*, 9642-9655.

S4. Schmitt, S., Evers, J.F., Duch, C., Scholz, M., and Obermayer, K. (2004). New methods for the computer-assisted 3-D reconstruction of neurons from confocal image stacks. Neuroimage *23*, 1283-1298.

S5. Evers, J.F., Schmitt, S., Sibila, M., and Duch, C. (2005). Progress in functional neuroanatomy: precise automatic geometric reconstruction of neuronal morphology from confocal image stacks. J Neurophysiol *93*, 2331-2342.

S6. Vactor, D.V., Sink, H., Fambrough, D., Tsoo, R., and Goodman, C.S. (1993). Genes that control neuromuscular specificity in *Drosophila*. Cell *73*, 1137-1153.

S7. Glicksman, M.A., and Brower, D.L. (1988). Expression of the Sex combs reduced protein in *Drosophila* larvae. Dev Biol *127*, 113-118.

S8. Takagawa, K., and Salvaterra, P. (1996). Analysis of choline acetyltransferase protein in temperature sensitive mutant flies using newly generated monoclonal antibody. Neuroscience Research *24*, 237-243.

S9. Wilson, R.I., and Laurent, G. (2005). Role of GABAergic inhibition in shaping odor-evoked spatiotemporal patterns in the *Drosophil*a antennal lobe. J Neurosci *25*, 9069-9079.

S10. Mahr, A., and Aberle, H. (2006). The expression pattern of the Drosophila vesicular glutamate transporter: a marker protein for motoneurons and glutamatergic centers in the brain. Gene Expr Patterns *6*, 299-309.

S11. Diegelmann, S., Bate, M., and Landgraf, M. (2008). Gateway cloning vectors for the LexA-based binary expression system in *Drosophila*. Fly (Austin) *2*.

S12. Bashaw, G.J., and Goodman, C.S. (1999). Chimeric axon guidance receptors: the cytoplasmic domains of slit and netrin receptors specify attraction versus repulsion. Cell *97*, 917-926.

S13. Pfeiffer, B.D., Ngo, T.T., Hibbard, K.L., Murphy, C., Jenett, A., Truman, J.W., and Rubin, G.M. (2010). Refinement of tools for targeted gene expression in *Drosophila*. Genetics *186*, 735-755.

S14. Ni, J.Q., Liu, L.P., Binari, R., Hardy, R., Shim, H.S., Cavallaro, A., Booker, M., Pfeiffer, B.D., Markstein, M., Wang, H., et al. (2009). A Drosophila resource of transgenic RNAi lines for neurogenetics. Genetics 182, 1089-1100.
